# Using Multilevel Mediation Model to Measure the Contribution of Beliefs to Judgments of Learning

**DOI:** 10.3389/fpsyg.2020.00637

**Published:** 2020-04-15

**Authors:** Xiao Hu, Jun Zheng, Tian Fan, Ningxin Su, Chunliang Yang, Liang Luo

**Affiliations:** ^1^Collaborative Innovation Center of Assessment Toward Basic Education Quality, Beijing Normal University, Beijing, China; ^2^Institute of Developmental Psychology, Beijing Normal University, Beijing, China

**Keywords:** metamemory, judgments of learning, beliefs, multilevel linear model, multilevel mediation model

## Abstract

Recent studies on judgments of learning (JOLs) suggest that beliefs play an important role in the formation of JOLs. The current article introduces a multilevel mediation model to quantify the contribution of beliefs to JOL formation when both JOLs and global belief-based predictions are measured from the same group of participants. Our examples of fitting the multilevel mediation model to hypothetical and published datasets revealed that it is feasible to use the multilevel mediation model to examine the mediation effect of beliefs on the relationship between a cue and JOLs, and quantitatively compare the effects of beliefs and processing fluency on JOLs in one model. Then we compared the current multilevel mediation model and the multilevel moderation model implemented in previous studies, and discussed their similarities and differences. Finally, a data simulation was performed to explain the inflation of Type I error for the multilevel mediation model when we regress global belief-based predictions on the cue, and suggestions about appropriate steps for conducting multilevel mediation analysis are proposed.

## Introduction

Judgment of learning (JOL) refers to metacognitive judgements regarding the likelihood of remembering given studied items in later memory tests (Dunlosky and Metcalfe, 2009; Rhodes, 2016), which is a critical component of metamemory monitoring. JOLs play an important role in self-regulated learning: people rely on JOLs to adjust their subsequent learning behaviors, such as the allocation of study time and the selection of items for restudy (Metcalfe and Kornell, 2005; Metcalfe and Finn, 2008; Yang et al., 2017). Thus, understanding how people form JOLs is of critical importance, and indeed the mechanisms underlying the formation of JOLs attract great attention from researchers (for a review, see Rhodes, 2016). According to the cue-utilization view (Koriat, 1997), people cannot directly monitor their memory strength when making JOLs. Instead, JOLs are inferential in nature and are based on a variety of cues, including the characteristics of the study items and the conditions of learning (Bjork et al., 2013).

In early studies, it was assumed that cues affect JOLs mainly through their effects on processing fluency during learning, which refers to the subjective experience of the ease or difficulty of information processing (Hertzog et al., 2003; Castel et al., 2007; Rhodes and Castel, 2008). Recently, Mueller et al. (2013, 2014b, 2016) and Mueller and Dunlosky (2017) demonstrated that people's beliefs about how given cues affect performance could also play an important role in their effects on JOLs. For example, Mueller et al. (2014b) examined the role of beliefs in the font-size effect on JOLs. Many studies have shown that people give higher JOLs for words presented in large than small font size, while font size does not significantly affect memory performance (Rhodes and Castel, 2008; Kornell et al., 2011; Susser et al., 2013; Hu et al., 2015; Yang et al., 2018). In their study, Mueller et al. measured participants' beliefs either using global predictions in belief questionnaires or the JOLs made before learning each word (i.e., pre-study JOLs; see Castel, 2008). The results revealed that participants had *a priori* beliefs that large words are easier to remember than small ones, suggesting that such beliefs may contribute to the font-size effect on JOLs. Following studies indicate that beliefs play an important role in the effect of different cues on JOLs, including font size, volume, semantic relatedness, word frequency, concreteness, and so on (Mueller et al., 2013, 2016; Hu et al., 2015; Jia et al., 2016; Frank and Kuhlmann, 2017; Li et al., 2017; Witherby and Tauber, 2017; Su et al., 2018).

In previous studies, researchers have used different methods to examine whether people's beliefs about memory performance significantly contribute to a given cue's effect on JOLs. Many studies measured the effect of cues on JOLs and beliefs in separate groups of participants, which documented that both JOLs and beliefs were significantly affected by the cues. Based on these results, they suggested that beliefs are at least one of the sources underlying the cue effect on JOLs (Mueller et al., 2013, 2014b, 2016; Jia et al., 2016; Witherby and Tauber, 2017).

However, simply investigating the effect of cues on both JOLs and beliefs in separate groups cannot make sure to what extent people utilize those beliefs to form their JOLs (Koriat et al., 2004; Kornell and Bjork, 2009; Kornell et al., 2011; Tauber et al., 2019). For example, Kornell et al. (2011) observed that when queried directly in a belief questionnaire, participants showed a metamemory belief that more study opportunities should produce superior memory performance. However, in another experiment, item-by-item JOLs were largely insensitive to future study opportunities. Based on these results, Kornell et al. suggest that participants may fail to use their beliefs about study opportunities when making online JOLs. Thus, it may be more appropriate to measure JOLs and beliefs from the same participant and examine whether beliefs could significantly predict JOLs when we aim to quantify the contribution of beliefs to the JOL process.

To further examine the effect of beliefs on JOLs within the same participants, some previous studies have asked one group of participants to first make global belief-based predictions about their memory and then to provide JOLs to study items (Ariel et al., 2014; Hu et al., 2015; Frank and Kuhlmann, 2017; Su et al., 2018; Schaper et al., 2019). In these studies, participants predict how many items they will recall in a future test before they start learning the items. This global prediction can only be based on participants' prior beliefs because they have not seen the items. Then during the learning phase, participants give a JOL after seeing each item. Researchers in these studies have used different statistical methods to investigate how much beliefs contribute to JOLs within the same participants. For example, Hu et al. (2015) asked participants to first make global predictions (before the learning phase) about what proportion of words presented in large or small font they would remember in a memory test. Then participants learned a list of large and small words, and gave a JOL to each word. To examine the role of beliefs in the font-size effect on JOLs, Hu et al. calculated the difference in belief-based predictions and mean JOLs between large and small words for each participant. Their regression analysis revealed that differences in beliefs could significantly predict the difference in mean JOLs between large and small words, suggesting that participants used their beliefs about font size in the JOL process.

This simple linear regression analysis can directly quantify the effect of beliefs on JOLs across participants. However, it unravels the relationship between beliefs and JOLs at the participant level while overlooking the data at the item level (i.e., JOL for each item). Whether beliefs contribute to the effect of cues on JOLs for each item is of actual interest, and it might be inappropriate to draw conclusions about to what extent cues affect JOLs through beliefs at a lower level (i.e., item level) based on data from a higher level (i.e., mean JOLs at the participant level) (Raudenbush and Bryk, 2002).

In addition, it is relatively difficult to use participant-level regression analysis to quantify and compare the contributions of beliefs and processing fluency in JOL formation. Previous studies have employed different measurements of processing fluency, such as study time in self-paced study, trials to acquisition, and response time in lexical or perceptual decision tasks (e.g., Mueller et al., 2014b; Undorf and Erdfelder, 2015; Yang et al., 2018), and fluency is often measured for each individual item. To quantify the contribution of processing fluency to JOLs at the item level, previous studies often employed a multilevel linear model to delineate the relationship between fluency and JOLs (Undorf and Erdfelder, 2015; Undorf et al., 2017; Yang et al., 2018). Thus, it should be better to also explore the role of beliefs in the JOL process at the item level if we want to compare the contribution of beliefs and processing fluency to JOLs (Frank and Kuhlmann, 2017; Su et al., 2018; Schaper et al., 2019). Researchers should use appropriate statistical methods to examine to what extent cues affect item-by-item JOLs through global beliefs about memory performance, and quantitatively compare the effect of beliefs and fluency on JOLs at the item level.

Here we introduce a multilevel mediation model to examine the role of beliefs in JOL process when both JOLs and global belief-based predictions are measured within the same group of participants. This model concerns whether beliefs significantly mediate the cue effect on JOLs at the item level. In the current article, we first introduce the mathematical equations of this multilevel mediation model. Then we show examples of how to use this multilevel mediation model to investigate the relationship between beliefs and JOLs in hypothetical and empirical datasets, and how to quantitatively compare the effect of beliefs and fluency on JOLs in one model. Next, we discuss the similarities and differences between the current multilevel mediation model and the multilevel moderation model used in previous studies (Frank and Kuhlmann, 2017; Schaper et al., 2019). Finally, we show the inflation of Type I error in the multilevel mediation model when global belief-based judgments are regressed on the cues, and offer suggestions about how to use this multilevel mediation model.

## The Multilevel Mediation Model

**Figure 1:**
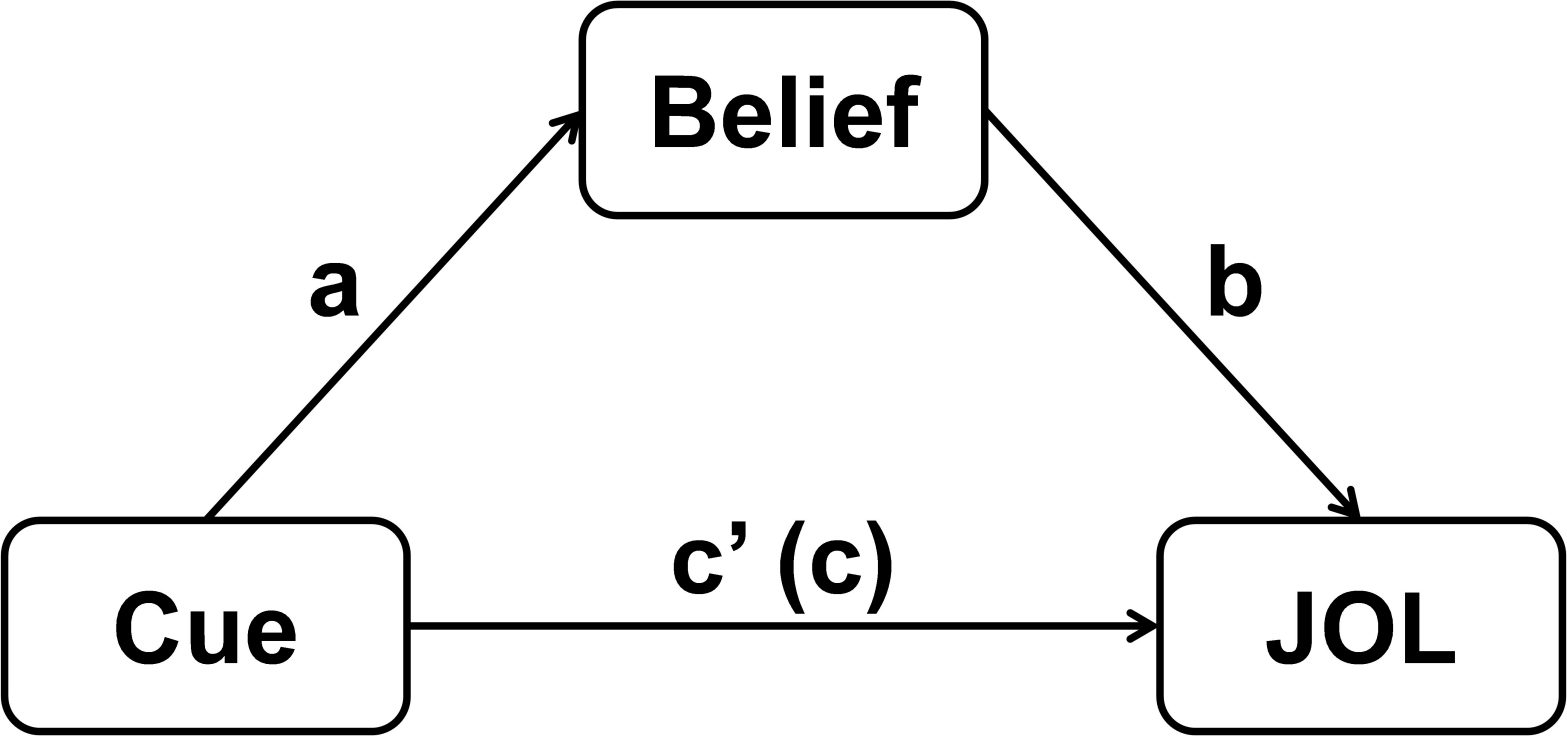
Schematic illustration of the mediation model for quantifying the contribution of beliefs to the cue effect on JOLs. The coefficient *c* represents the total effect of cue on JOLs, and *c'* represents the direct effect of cue on JOLs when the effect of beliefs is controlled.

A mediation model concerns whether a mediator variable can significantly account for the relationship between a predictor variable and an outcome variable (Baron and Kenny, 1986). Similarly, the multilevel mediation model introduced here focuses on whether participants' beliefs about how a cue affects memory performance (typically measured by global belief-based predictions) mediate the cue effect on JOLs at the item level. To build a mediation model, we need to first regress the mediator variable (i.e., beliefs) on the predictor variable (i.e., cue), and then regress the outcome variable (i.e., JOLs) on both the predictor and mediator variables (Baron and Kenny, 1986). See Figure 1 for the explanation of the mediation model.

In this section, we will explain the multilevel mediation model based on the data from a hypothetical experiment. The data are included in the file *example_data.csv* which can be downloaded from the OSF repository (https://osf.io/dsnj6/). There are three participants in this hypothetical experiment, and each participant learns six words and gives a JOL for each word. Words are divided into two experimental conditions that differ in the level of a cue (e.g., font size). Three words belong to the condition of Cue Level 1 (e.g., large font) and the other three words are from the condition of Cue Level 2 (e.g., small font). For the third participant in this experiment, the JOL is missing for one trial with Cue Level 2 and this trial is excluded from the data analysis (i.e., the data file only includes five trials for the third participant).

Before the learning phase, participants predict the overall proportion of recalled words in a future memory test based on their prior beliefs. They give global predictions separately for Cue Levels 1 and 2 in a questionnaire. In addition, the learning process is self-paced and the study time for each word is also recorded.

There are five variables in the data file *example_data.csv*, including *SubID* (the ID for each participant), *Cue* (the cue levels, in which we code Cue Level 1 as 1 and Cue Level 2 as −1), *Belief* (global belief-based predictions for the overall proportions of recalled trials for Cue Levels 1 and 2), *ST* (self-paced study time in seconds), and *JOL* (on a percentage scale from 0 to 100).

We aim to build a multilevel mediation model for this hypothetical dataset to examine the mediation effect of beliefs on the relationship between the cue levels and JOLs. First, we should regress beliefs for each item on the variable *Cue*. The equations are as follows:

(1) $$\begin{array}{l} Belief_{ij}=\beta_{0j\left(1\right)}+a_{j}Cue_{ij}+\varepsilon_{ij\left(1\right)} \end{array}$$

(2) $$\begin{array}{l} \beta_{0j\left(1\right)}=r_{00\left(1\right)}+\mu_{0j\left(1\right)} \end{array}$$

(3) $$\begin{array}{l} a_{j}=a \end{array}$$

In the equations, *i* represents the trial within each participant, and *j* denotes each participant. The *Cue*_*ij*_ is the level of the cue for each trial. In the hypothetical dataset, the value of *Cue*_*ij*_ is equal to 1 for Cue Level 1 and −1 for Cue Level 2. The *Belief*_*ij*_ is the belief-based prediction of memory performance for each trial. When beliefs are measured with global predictions, each participant only gives one belief-based prediction of memory performance for each level of the cue (Hu et al., 2015; Frank and Kuhlmann, 2017; Su et al., 2018; Schaper et al., 2019). Thus, for each participant, all of the items within the same level of the cue have the same value of belief-based prediction. For example, the belief-based predictions for the first participant in the hypothetical dataset are 60% for all trials with Cue Level 1 and 30% for all trials with Cue Level 2. This is because the first participant makes global belief-based predictions that he or she will recall 60% of the words with Cue Level 1 and 30% of the words with Cue Level 2 in a future memory test. Although the belief-based predictions remain the same within the same level of the cue, there is still variability for the variable *Belief* across cue levels for each participant. Thus, in the current multilevel mediation model, we treat *Belief* as a variable at the item level (rather than at the participant level).

In the equations above, β_0*j*(1)_ represents the intercept in the regression model for each participant, which is the sum of a fixed intercept *r*_00(1)_ (same for all participants) and a random intercept μ_0*j*(1)_ (different across participants). The *a*_*j*_ represents the effect of path *a* for each participant (see Figure 1), which is the effect of predictor variable (*Cue*) on the mediator variable (*Belief* ). Finally, ε_*ij*(1)_ represents the residual error for each trial in the regression model.

Here we fix the slope *a*_*j*_ as the same value for all participants [i.e., *a* in Equation (3)] and does not add the random slope. This is because, in many previous studies (and in the hypothetical dataset), the predictor variable *Cue* only contains two levels (e.g., large and small font size) and the mediator variable *Belief* is the same within each level of the cue for a participant (Hu et al., 2015; Frank and Kuhlmann, 2017; Su et al., 2018; Schaper et al., 2019). In this case, the random effect for slope *a*_*j*_ is perfectly confounded with the residual error, which can make the model fail to converge (Singmann and Kellen, 2020) (see section “Further Explanation About the Random Slope of Path *a* When We Regress Beliefs on the Cue in Multilevel Mediation Model” in the Supplementary Material for further explanation on this topic).

It is definitely true that the effect of cue on belief-based predictions is different across participants, because the difference in beliefs across cue levels is higher for some participants than the others. However, in order to make the model converge, we can only assume that the cue effect on beliefs is fixed in the multilevel mediation model, and estimate an overall effect of cue on beliefs for all participants. This is similar to the paired *t*-test used for examining the effect of a cue (with two levels) on belief-based predictions at the participant level, which has been applied in many previous studies (e.g., Mueller et al., 2014b; Hu et al., 2015; Frank and Kuhlmann, 2017). Although the difference in beliefs between the cue levels varies across participants, we can only examine whether the overall difference in beliefs between two levels of the cue significantly differs from 0 for a group of participants.

Next, we need to regress JOLs on the cue and belief-based predictions. The equations are as follows:

(4) $$\begin{array}{l} JOL_{ij}=\beta_{0j\left(2\right)}+b_{j}Belief_{ij}+{c^{\prime }}_{j}Cue_{ij}+\varepsilon_{ij\left(2\right)} \end{array}$$

(5) $$\begin{array}{l} \beta_{0j\left(2\right)}=r_{00\left(2\right)}+\mu_{0j\left(2\right)} \end{array}$$

(6) $$\begin{array}{l} b_{j}=b+\mu_{bj} \end{array}$$

(7) $$\begin{array}{l} {c^{\prime }}_{j}=c^{\prime }+\mu_{c^{\prime }j} \end{array}$$

In the equations above, *JOL*_*ij*_ denotes the JOL value for each trial. The β_0*j*(2)_ represents the intercept for each participant, which is the sum of a fixed intercept *r*_00(2)_ and a random intercept μ_0*j*(2)_. The slopes *b*_*j*_ and *c'*_*j*_ represent the effect of path *b* and *c'* in the mediation model for each participant (see Figure 1). Path *b* is the effect of mediator variable (*Belief* ) on the outcome variable (*JOL*), and path *c'* is the direct effect of the predictor variable (*Cue*) on the outcome variable when the effect of mediator is controlled. Unlike path *a*, we should add the random slopes for path *b* and *c* into the model (Barr et al., 2013).

The indirect effect of cue on JOLs through beliefs, or the mediation effect of beliefs on the relationship between the cue and JOLs, can be computed using the following equation (Rockwood and Hayes, 2017):

(8) $$\begin{array}{l} IND_{belief}=ab+\sigma_{a_{j}b_{j}} \end{array}$$

In this equation, *a* represents the fixed effect for path *a* (the cue effect on beliefs) and *b* represents the fixed effect for path *b* (the belief effect on JOLs). The σ_*ajbj*_ represents the covariance between the regression coefficients of path *a* and *b* for each participant (i.e., *a*_*j*_ and *b*_*j*_). If the random slope is added for the path *a* and *b*, then the regression coefficient for each path should vary across participants, and there may be a covariance between the regression coefficients for the two paths. However, in the hypothetical dataset (and in many previous studies), we need to remove the random slope for path *a* to make the model converge. In this case, the regression coefficient for path *a* is the same across all participants, and there is no covariance between the regression coefficients for path *a* and *b* (i.e., σ_*ajbj*_ = 0).

The proportion of the cue effect on JOLs mediated by beliefs is equal to the indirect effect divided by the total effect (the sum of indirect and direct effect of the cue):

(9) $$\begin{array}{l} Prop_{med}=\frac{IND_{belief}}{IND_{belief}+c^{\prime }}=\frac{ab+\sigma_{a_{j}b_{j}}}{ab+\sigma_{a_{j}b_{j}}+c^{\prime }} \end{array}$$

The mediation effect in the multilevel mediation model (i.e., the indirect effect of cue on JOLs through beliefs) can be divided into within-participant and between-participant mediation effects. The within-participant mediation effect refers to whether the mediator variable (*Belief* ) for each trial can mediate the relationship between the predictor (*Cue*) and outcome variable (*JOL*) for the same trial within each participant. In contrast, the between-participant mediation effect refers to the mediation effect at the participant level: we can calculate the mean of the variables *Cue, Belief* and *JOL* for all trials (including all levels of the cue) separately for each participant, and the between-participant mediation effect represents whether the mean of *Belief* can mediate the relationship between the mean of *Cue* and mean of *JOL* across participants (Zhang et al., 2009). The within-participant mediation effect is of main interest when we investigate the role of beliefs in the JOL process, because (a) we aim to examine whether beliefs contribute to the cue effect on JOLs within each participant, and (b) the between-participant mediation effect focuses on the averaged variables for all trials including all levels of the cue, which is typically not of interest.

To estimate the within-participant mediation effect, we need to group-mean-center the predictor variable *Cue* and the mediator variable *Belief* (Vuorre and Bolger, 2018). Group-mean centering refers to centering the variable around the mean of each group at the high level in the multilevel linear model, which is the mean for each participant in the current analysis. To perform the group-mean centering for a variable, we should first calculate the mean for each participant and then subtract each participant's mean from the score of each trial within the same participant.

When the variable *Cue* only has two levels (e.g., Cue Level 1 and Cue Level 2, such as large and small sizes) and the trial numbers for the two levels are the same for each participant, we only need to code Cue Level 1 (e.g., large font) as 1 and Cue Level 2 (e.g., small font) as −1 to make the variable *Cue* group-mean-centered for each participant because the mean of the variable *Cue* for each participant is 0. However, if the number of trials for two levels of the cue is different (e.g., when there are missing data in some trials for one of the cue levels), we need to subtract the averaged variable *Cue* for all trials within each participant from the value of *Cue* for each trial.

For example, we code Cue Level 1 as 1 and Cue Level 2 as −1 in the hypothetical dataset, and for the first two participants the number of trials is the same for the two levels of the cue. Thus, the variable *Cue* has already been group-mean-centered for the first two participants because the mean of *Cue* for all trials within each participant is 0. However, for the third participant, there are 3 trials for Cue Level 1 and 2 trials for Cue Level 2. The mean of the variable *Cue* for five trials is 0.2 for the third participant, and after we perform the group-mean centering, the centered variable *Cue* should be equal to 1–0.2 = 0.8 for Cue Level 1 and (−1)−0.2 = −1.2 for Cue Level 2.

Similarly, to group-mean-center the variable *Belief* , we should subtract the averaged *Belief* for all trials (including the Cue Levels 1 and 2) within each participant from the value of *Belief* for each trial. For example, for the first participant in the hypothetical dataset, the belief-based predictions of overall recall proportion are 60% for Cue Level 1 and 30% for Cue Level 2. In addition, there are 3 trials for Cue Level 1 and 3 trials for Cue Level 2. Thus, the mean of the variable *Belief* for all trials is (60% × 3 + 30% × 3)/(3 + 3) = 45%, and the centered variable *Belief* is equal to 15% for each trial with Cue Level 1 and −15% for each trial with Cue Level 2. For the third participant, the variable *Belief* is 70% for Cue Level 1 and 60% for Cue Level 2, and there are 3 trials for Cue Level 1 and 2 trials for Cue Level 2. Thus, the mean of *Belief* is (70% × 3 + 60% × 2)/(3 + 2) = 66%, and the centered variable *Belief* is 4% for Cue Level 1 and −6% for Cue Level 2.

We should also note that when the variables *Cue* and *Belief* are both group-mean-centered, we need to remove the random intercept when *Belief* was regressed on *Cue* at the item level because the mean of centered belief-based predictions for each participant is always zero and does not vary across participants.

In the multilevel mediation model, it is feasible to simultaneously examine the contribution of beliefs and processing fluency to JOLs at the same time. We only need to add two mediator variables (beliefs and fluency) in the model and compare the effects of the two mediators. For example, the self-paced study time in the hypothetical dataset can be seen as a measurement of processing fluency, and we can add both the belief-based predictions and study time into the model as two mediators. In order to estimate the within-participant mediation effect of fluency, we need to group-mean-center the processing fluency for each participant.

When we regress the centered processing fluency on the centered variable *Cue* (i.e., path *a*), we should remove the random intercept because the mean of centered fluency is always zero for each participant. However, we need to add the random slope when processing fluency is regressed on the cue. This is different from the cue effect on beliefs, in which we must remove the random slope to make the model converge. The reason for this difference is that processing fluency is often measured for each trial using response time or self-paced study time (e.g., Yang et al., 2018; Schaper et al., 2019), and is different across trials within a certain level of the cue for each participant (as in the hypothetical dataset). When we regress processing fluency on the cue, the model can converge when the random slope is added, which accounts for the variability in the cue effect on fluency across participants. Furthermore, when we regress JOLs on the cue levels, beliefs and fluency, we should include all of the random intercept and random slopes into the model (Barr et al., 2013).

## Using the Multilevel Mediation Model: A Practical Example

In this section, we provide a practical example of how to use the multilevel mediation model to examine the mediation effect of beliefs on the relationship between a cue and JOLs, and to compare the effect of beliefs and processing fluency on JOLs. We fit the multilevel mediation model to the hypothetical data from *example_data.csv* introduced in previous section. We aim to first examine the mediation effect of beliefs on the relationship between the cue and JOLs, and then compare the mediation effect of beliefs and processing fluency (measured with self-paced study time).

In order to estimate the within-participant mediation effect of beliefs and study time, we need to first group-mean-center the cue levels, belief-based predictions and study time. We can manually calculate the mean of these variables for each participant, and then subtract each participant's mean from the score of each trial. An easier way to perform the group-mean centering is to use the *isolate* function from the *bmlm* package in R, which can automatically output the centered variables (Vuorre and Bolger, 2018). We can set the working directory in R as the directory containing the data file, and run the following code:

example_data  <- read.csv (“example_data.csv”)

library (bmlm)

example_data <- isolate(d = example_data, by =

“SubID,” value = c (“Cue,”“Belief,” “ST”))

The first line of the code above inputs the data into the workspace in R. The second line loads the *bmlm* package (needed to be installed first). The third line uses the *isolate* function to group-mean-center the variables. In the *isolate* function, the argument *d* represents the name of a data frame in R, *by* represents the variable name for subject ID, and *value* refers to the variables for group-mean centering, in which the names of multiple variables should be connected using the *c* function. The *isolate* function creates a centered variable for each of the variables in *value*, and the new centered variables are labeled with “_cw” (e.g., *Cue_cw, Belief_cw* and *ST_cw*).

Next, we perform the multilevel mediation analysis with the MLmed macro in SPSS (Rockwood and Hayes, 2017). We need to first export the data frame in R (*example_data*) as a data file for SPSS (e.g., *example_data.sav*), which is based on the *write_sav* function in the *haven* package (needed to be installed first). We should run the following code in R:

library (haven)

write_sav (example_data,”example_data.sav”)

The Beta 2 version of the MLmed macro can be downloaded from its official website (https://njrockwood.com/mlmed). After opening the data file *example_data.sav* in SPSS, we should run the syntax *MLMED_Beta_2.sps* in the MLmed package to load the MLmed macro into SPSS. We need to open *MLMED_Beta_2.sps* in a syntax window of SPSS, and choose **Run - All** from the menu in the syntax window to run this syntax.

We then perform a multilevel mediation analysis to examine the mediation effect of beliefs on the relationship between the cue and JOLs. We can open a new syntax window in SPSS and write the following syntax:

MLmed data = DataSet1

/x = Cue_cw

/m1 = Belief_cw 

/y = JOL 

/cluster = SubID 

/randx = 10

/randm = 1 

/xB = 0

/mB = 0 

/randMint = 0

/folder = D:\mlmed_temp\.

In the syntax above, *data* refers to the dataset name, which should be the name that appears in brackets on the opened dataset window, rather than name for the *.sav* file. By default, the first dataset opened in a new SPSS session is named *DataSet1*. The */x* represents the name of the predictor variable, and here we use the centered cue levels (i.e., *Cue_cw*). The */m1* represents the name of the mediator variable, which should be the centered belief-based predictions (i.e., *Belief_cw*). The */y* refers to the outcome variable, and should be the JOLs. The */cluster* represents the variable name for subject ID (i.e., *SubID*).

The */randx* represents whether the random slope for the predictor variable (*Cue_cw*) is added into the model, and contains two numbers. The first number refers to the random slope for path *c'*, which is the direct effect of the predictor variable on the outcome variable (*JOL*). The second number refers to the random slope for path *a*, which is the effect of predictor variable on the mediator variable (*Belief_cw*). Here we set */randx* = 10 to add the random slope for path *c'*, but remove the random slope for path *a* to make the model converge [see the discussion after Equation (3)]. The */randm* represents the random slope for the effect of mediator variable (*Belief_cw*) on the outcome variable (*JOL*), and we set */randm* = 1 to add this random slope.

The */xB* refers to whether there is between-participant effect of the predictor on the mediator variable, and */mB* represents whether there is between-participant effect of the mediator on the outcome variable. Here we set both */xB* and */mB* as 0 because the variables *Cue_cw* and *Belief_cw* have been group-mean-centered and there is no between-participant variance for these variables. In addition, */randMint* represents whether the random intercept should be added when the mediator variable (*Belief_cw*) is regressed on the predictor variable (*Cue_cw*). We set */randMint* = 0 to remove this random intercept because the mean of the centered belief-based predictions should always be zero for each participant and thus does not vary across participants^1^. Finally, */folder* refers to the name of the folder that stores the temporary results from MLmed (the folder must be created first).

When we run the syntax above, we will receive the following error message:


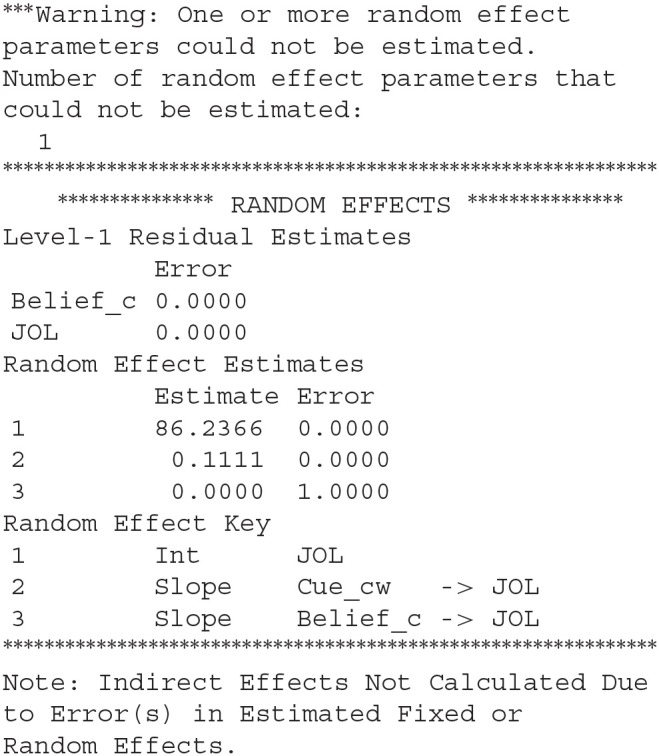


This error message suggests that the third random effect, which is the random slope for the effect of *Belief_cw* on JOLs, cannot be estimated by SPSS, suggesting that there might be no random effect for this slope in the current dataset. Thus, we have to remove this random slope before estimating the parameters in the model. Our syntax needs to be revised as follows:

MLmed data = DataSet1 

/x = Cue_cw

/m1 = Belief_cw 

/y = JOL 

/cluster = SubID 

/randx = 10

/randm = 0 

/xB = 0

/mB = 0 

/randMint = 0

/folder = D:\mlmed_temp\.

The only difference between the current and previous syntax is that here we set */randm* = 0 to remove the random slope for the effect of mediator (*Belief_cw*) on the outcome variable (*JOL*). We should note that whether we need to remove the random slope for the belief effect on JOLs may differ across different datasets. We recommend that we should always start with adding all of the random intercept and slopes when JOLs are regressed on the cue and beliefs, and then only remove the random slopes that cannot be estimated by SPSS (Barr et al., 2013).

After we run the new syntax, results from MLmed will be shown in the output window of SPSS. There are a lot of different results and we only introduce the ones we are mostly interested in. The first important result is the fixed effects in the multilevel linear model:


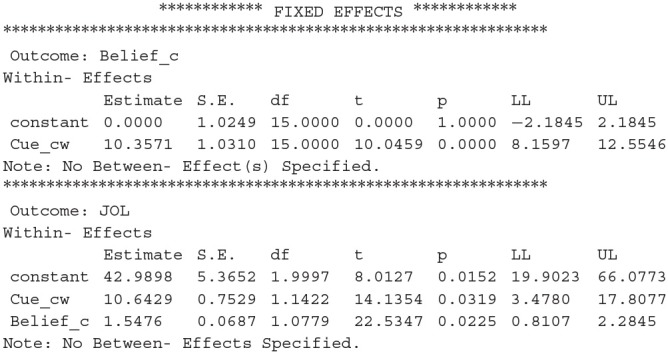


The first part of the results shows the coefficients for the regression of *Belief_cw* on *Cue_cw*, and the second part shows the coefficients for the regression of *JOL* on *Cue_cw* and *Belief_cw*. We can conclude that cue levels have a significant effect on beliefs, and JOLs are significantly predicted by both the cue levels and beliefs. The LL and UL represent the lower and upper bound for 95% confidence interval (CI).

The second important result is the direct and indirect effect of the cue on JOLs:


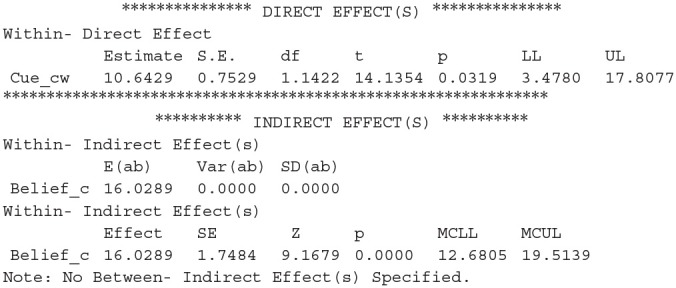


The results above reveal that the direct effect of cue on JOLs is significant, which is equal to the regression coefficient for the effect of *Cue_cw* on JOLs (i.e., path *c'* in Figure 1). Thus, the cue levels can significantly predict JOLs when the effect of beliefs is controlled. In addition, the indirect effect of cue on JOLs through beliefs is also significant, suggesting that beliefs significantly contribute to the cue effect on JOLs. The MCLL and MCUL are the lower and upper bound of 95% CI estimated with Monte Carlo method, and may be slightly different each time we run the syntax. We can then compute the proportion of the cue effect on JOLs mediated by beliefs, which is equal to 16.0289/(10.6429+16.0289) = 60.1%.

We then add the centered study time (*ST_cw*) into the model as another mediator, and compare the mediation effect of beliefs and study time on JOLs. The syntax is written as follows:

MLmed data = DataSet1

/x = Cue_cw

/m1 = Belief_cw 

/m2 = ST_cw 

/y = JOL 

/cluster = SubID 

/randx = 101

/randm = 01 

/xB = 0

/mB = 00 

/randMint = 00 

/folder = D:\mlmed_temp\.

In the syntax above, */m1* and */m2* represent the name for the first and second mediator variable. The */randx* represents whether we add the random slope for the effect of predictor variable (*Cue_cw*) on the outcome variable (*JOL*), the first mediator (*Belief_cw*) and the second mediator (*ST_cw*). We set */randx* = 101 to remove the random slope for the cue effect on beliefs (as in previous analysis) and add the random slope for the cue effect on JOLs and study time. We add the random slopes when study time is regressed on the cue levels because study time is different across trials within a certain level of the cue for a participant, and the model with random slope can converge when we regress study time on the cue levels.

The */randm* refers to whether the random slope is added for the effect of first mediator (*Belief_cw*) and second mediator (*ST_cw*) on the outcome variable (*JOL*). We set */randm* = 01 to add the random slope for the effect of study time on JOLs but remove the random slope for the belief effect on JOLs which cannot be estimated by SPSS (as shown in previous analysis). We also recommend that we should always start with adding all of the random intercept and random slopes when JOLs are regressed on the cue levels, beliefs and processing fluency, and then only remove the random slopes that cannot be estimated by SPSS. In addition, we set */xB* = 0 to remove the between-participant effect of the predictor variable (*Cue_cw*), */mB* = 00 to remove the between-participant effect of the first and second mediator (*Belief_cw* and *ST_cw*), and */randMint* = 00 to remove the random intercepts when the first and second mediator is regressed on the predictor variable, because the mean of the centered mediators is always zero for each participant and does not vary across participants.

The results from MLmed are shown in the output window of SPSS after we run the syntax. We first look at the fixed effects:


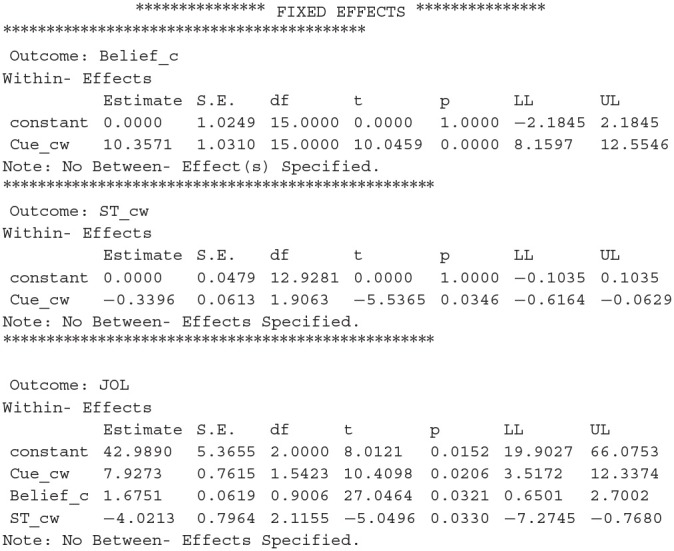


The results above show that cue levels have a significant effect on beliefs and study time, and JOLs are significantly predicted by the cue levels, beliefs and study time. In addition, adding the study time into the model slightly changes the regression coefficient for the belief effect on JOLs.

We then look at the direct and indirect effect of the cue on JOLs:


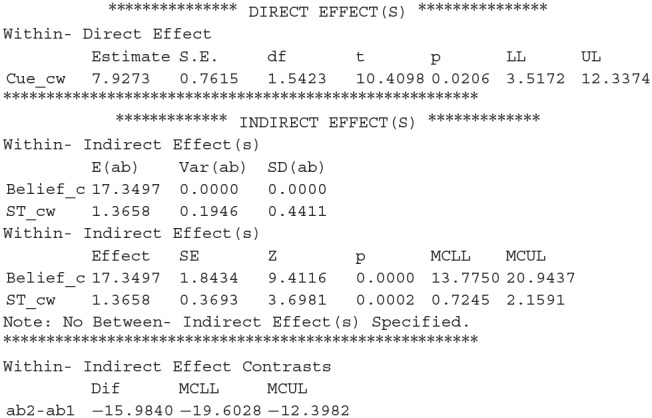


We can conclude that the mediation effects of beliefs and study time on JOLs are both significant, and the direct effect of cue on JOLs is also significant when the effect of the two mediator variables is controlled. We can then compute the proportion of the cue effect on JOLs mediated by each mediator variable, which is equal to 17.3497/(17.3497 + 1.3658 + 7.9273) = 65.1% for beliefs and 1.3658/(17.3497 + 1.3658 + 7.9273) = 5.1% for study time. The estimation of mediation proportion for beliefs slightly changes when study time is added into the model as another mediator, due to the slight change in the regression coefficient of belief effect on JOLs when study time is added in the regression.

The last result shows the comparison between the two mediation effects, in which *ab2* and *ab1* represent the mediation effect for the second and first mediator (*ST_cw* and *Belief_cw*), respectively. We can see that the mediation effect of study time is significantly lower than that of beliefs because the 95% CI for the difference does not contain zero.

## Fitting Multilevel Mediation Model to Published Data

In this section, we show an example of fitting the multilevel mediation model to empirical datasets from previous studies to examine to what extent cues affect item-by-item JOLs through global beliefs about memory performance. We fitted multilevel mediation models to datasets from eight previously published experiments, including Experiment 2 in Hu et al. (2015), Experiments 1–3 in Frank and Kuhlmann (2017), Experiments 2a and 2b in Su et al. (2018), and Experiments 1–2 in Schaper et al. (2019). Hu et al. and Su et al. investigated the role of beliefs in the font-size effect on JOLs. Frank and Kuhlmann explored whether beliefs contributed to the volume effect on JOLs. Schaper et al. examined the contribution of beliefs to the effect of expectancy (i.e., whether the study item is a typical item in a room) on JOLs. We chose these eight experiments because these experiments measured each participant's global belief-based predictions and item-by-item JOLs at the same time. In all of these eight experiments, participants first made global belief-based predictions about memory performance and then made JOLs for words with different levels of a cue (font size, volume, or expectancy). There are only two levels of the cue in each experiment (large and small font size/volume, or expected/unexpected item).

In Frank and Kuhlmann‘s (2017) Experiments 2 and 3, participants were randomly allocated into either a large-dose and a small-dose group, and the difference in dose between large and small volume was different in the two groups. Therefore, we treated the large and small dose conditions as independent experiments, and analyzed the data separately. In Schaper's et al. (2019) Experiments 1 and 2, participants were asked to give two predictions for each word during the learning phase, including a JOL for item memory and another prediction for source-memory performance (termed as judgment of source, or JOS). We separately analyzed the mediation effect of beliefs on the relationship between expectancy and two judgments (JOLs and JOSs).

In Schaper's et al. (2019) Experiment 2, the learning phase was self-paced and processing fluency for each item was measured with self-paced study time. Thus, we also performed another multilevel mediation analysis for Schaper's et al. (2019) Experiment 2, in which we quantitatively examined and compared the mediation effects of beliefs and fluency on the relationship between expectancy and two judgments (JOLs and JOSs). We separately built a multilevel mediation model for JOLs and JOSs.

### Information of Published Experiments

#### Participants

There were 25 participants in Hu et al.'s (2015) Experiment 2. For Su et al. (2018), there were 30 participants in each of their Experiments 2a and 2b. In Frank and Kuhlmann (2017), there were 52, 135 (69 in the small-dose group and 66 in the large-dose group) and 87 (44 in the small-dose group and 43 in the large-dose group) participants in their Experiments 1–3, respectively^2^. For Schaper et al. (2019), there were 96 participants in Experiment 1 and 120 participants in Experiment 2.

#### Materials

The study materials were 40 Chinese two-character words in Hu et al. (2015) and Su et al. (2018). In Su et al., four words were used as either primary or recency buffer words and were excluded from all analyses. The words were randomly divided into two sets, and one set of words was presented in small font size (9-pt) and the other set in large font size (70-pt). The study materials in Frank and Kuhlmann (2017) were 50 English nouns. For each participant, half of the words were randomly assigned to the large volume condition and the other half to the small volume condition. The study materials in Schaper et al. (2019) were three lists of 32 nouns. Each list contained 16 typical items from kitchen and 16 items from bathroom. Participants saw items in two lists with room labels during the learning phase. Half of the study items were from the expected room and half were from the unexpected room. Another list was used as distractors in the memory test.

#### Procedure

In all of the experiments, participants first made global belief-based predictions about memory performance. Participants read a description about the memory task they would perform, and predicted their memory performance for words presented in each level of a cue (large and small font size/volume, or expected/unexpected item). They then learned a list of words and made item-by-item JOL for each word. Following the learning phase, participants took a memory test. In addition, the learning phase was self-paced in Schaper's et al. (2019). Experiment 2 and study time was measured for each item as processing fluency.

#### Data Analysis

We fitted multilevel mediation models to the data in these experiments to investigate whether beliefs could significantly mediate the relationship between cues and JOLs. In each experiment, we coded the predictor variable *Cue* as 1 for trials with large font/large volume/expected items, and −1 for small font/small volume/unexpected items. In Frank and Kuhlmann (2017) and Su et al. (2018), there were missing JOL values or JOL values higher than 100 for some trials. These trials were excluded, and the cue levels were then group-mean-centered. We also group-mean-centered the belief-based predictions in all experiments. In Schaper et al. (2019), we used the global belief-based predictions for item memory performance when we performed the multilevel mediation analysis for JOLs, and beliefs for source memory performance when we performed the analysis for JOSs.

The multilevel mediation model was fitted using the MLmed macro in SPSS (Rockwood and Hayes, 2017). When regressing participants' belief-based predictions on the cue levels (path *a* in Figure 1), we removed the random intercept because the belief-based predictions were group-mean-centered, and the mean of beliefs for all trials should always be zero for each participant. We also removed the random slope for cue effect on beliefs, because the belief-based predictions are the same for all trials within a certain level of the cue for a participant, and adding the random slope for cue effect on beliefs can make the model fail to converge [see our discussion after Equation (3)]. When regressing JOLs on the cue levels and beliefs, we included all of the random effects into the model, including the random intercept and random slopes for the effect of cue levels (path *c'*) and beliefs (path *b*) on JOLs (except when a random slope could not be estimated by SPSS, suggesting that there might be no random effect for the slope) (Barr et al., 2013). In addition, we set the covariance structure for the random effects as diagonal (which is the default covariance structure in MLmed) to remove the correlation between random effects, which often made the model fail to converge (Singmann and Kellen, 2020).

For Schaper's et al. (2019) Experiment 2, we then built another multilevel mediation model in which two mediator variables were added into the model: belief-based predictions and self-paced study time. Following Schaper et al. (2019), we eliminated trials in which self-paced study time was shorter than 200 ms or longer than a participant's individual mean plus three standard deviations. We then logarithmized study times to render the distribution closer to normality, and group-mean-centered the cue levels, belief-based predictions and study time. When we regressed belief-based predictions or self-paced study time on the cue levels, we removed the random intercepts because the mean of the two centered mediator variables was always zero for each participant. We also removed the random slope for the cue effect on beliefs (as in previous analysis). We should note that the random slope for the effect of cue on study time should be added into the model (which is different from the cue effect on beliefs) because study time was different across trials within a certain level of the cue for a participant, and the model could converge when the random slope was added. When we regressed JOLs on the cue levels, beliefs and study time, we included all of the random intercept and random slopes into the model, and then removed the random slopes that could not be estimated by SPSS.

In all of the analyses above, restricted maximum likelihood (REML) estimation was employed to estimate the parameters in the models (which is the default estimation method for MLmed) because REML could produce unbiased parameter estimation in a multilevel linear model (Harville, 1977).

### Results and Discussion

#### The Mediation Effect of Beliefs on JOLs

In the multilevel mediation model examining the mediation effect of beliefs on JOLs, two results are typically of interest. The first is the indirect effect of cue on JOLs through beliefs (i.e., *IND*_*belief*_), which reflects whether beliefs could significantly mediate the cue effect on JOLs. The second is the direct effect of cue on JOLs (path *c'* in Figure 1), which refers to the cue effect on JOLs when the effect of beliefs is controlled. Table 1 shows the results from the multilevel mediation model for each experiment (for Schaper's et al., 2019 Experiment 2, here we only added beliefs, but not self-paced study time, as the mediator variable). The indirect effect of cue on JOLs through beliefs was significant in Hu et al.'s (2015) Experiment 2, Frank and Kuhlmann‘s (2017) Experiments 1 and 2, Su et al.'s (2018) Experiment 2b and the JOS condition in Schaper's et al. (2019) Experiment 1, suggesting that beliefs played an important role in the formation of JOLs in these experiments. In addition, the direct effect of cue on JOLs was significant in the large-dose group of Experiment 2 and both groups of Experiment 3 in Frank and Kuhlmann (2017), Experiment 2a in Su et al. (2018) and Experiments 1 and 2 in Schaper et al. (2019), indicating that other factors (such as processing fluency) might also contribute to JOLs when the effect of beliefs was controlled.

**Table 1:** The results from the multilevel mediation model fitted to published datasets.

**Effect**	**Estimate (β)**	***SE***	***df***	***t* or *z* value**	***p*-value**	**95% CI**
**Hu et al. (****2015****), Experiment 2**						
*a*	7.60	0.19	998	40.73	<0.001	[7.23, 7.97]
*b*	0.33	0.12	29.88	2.81	0.009	[0.09, 0.57]
*c'*	1.56	1.12	288.70	1.39	0.165	[−0.64, 3.76]
*IND_*belief*_*	2.52	0.90		2.80	0.005	[0.77, 4.30]
**Frank and Kuhlmann (****2017****), Experiment 1**						
*a*	10.11	0.23	2572	44.90	<0.001	[9.66, 10.55]
*b*	0.20	0.08	54.07	2.44	0.018	[0.04, 0.37]
*c'*	1.48	0.78	545.96	1.90	0.058	[−0.05, 3.00]
*IND_*belief*_*	2.06	0.85		2.44	0.015	[0.41, 3.75]
**Frank and Kuhlmann (****2017****), Experiment 2, large–dose group**						
*a*	8.60	0.17	3254	51.60	<0.001	[8.28, 8.93]
*b*	0.30	0.07	63.67	4.54	<0.001	[0.17, 0.43]
*c'*	3.12	0.84	63.88	3.71	<0.001	[1.44, 4.80]
*IND_*belief*_*	2.57	0.57		4.52	<0.001	[1.45, 3.66]
**Frank and Kuhlmann (****2017****), Experiment 2, small–dose group**						
*a*	7.63	0.15	3410	51.55	<0.001	[7.34, 7.92]
*b*	0.16	0.08	43.32	2.05	0.047	[0, 0.31]
*c'*	0.38	0.67	51.71	0.57	0.570	[−0.96, 1.72]
*IND_*belief*_*	1.19	0.58		2.05	0.041	[0.04, 2.33]
**Frank and Kuhlmann (****2017****), Experiment 3, large-dose group**						
*a*	8.84	0.12	2118	72.37	<0.001	[8.06, 9.08]
*b*	−0.03	0.10	41.40	−0.32	0.754	[−0.23, 0.17]
*c'*	2.92	1.04	41.39	2.81	0.008	[0.82, 5.02]
*IND_*belief*_*	−0.27	0.88		−0.32	0.752	[−2.03, 1.42]
**Frank and Kuhlmann (****2017****), Experiment 3, small-dose group**						
*a*	8.25	0.20	2159	42.14	<0.001	[7.87, 8.64]
*b*	−0.10	0.06	41.25	−1.65	0.107	[−0.22, 0.02]
*c'*	2.28	0.75	41.76	3.03	0.004	[0.76, 3.80]
*IND_*belief*_*	−0.83	0.51		−1.65	0.100	[−1.84, 0.16]
**Su et al. (****2018****), Experiment 2a**						
*a*	5.86	0.25	1072	23.86	<0.001	[5.38, 6.34]
*b*	0.21	0.15	27.95	1.41	0.171	[−0.10, 0.52]
*c'*	4.26	1.49	28.05	2.86	0.008	[1.21, 7.31]
*IND_*belief*_*	1.24	0.88		1.40	0.161	[−0.50, 2.97]
**Su et al. (****2018****), Experiment 2b**						
*a*	6.77	0.13	1073	50.22	<0.001	[6.50, 7.03]
*b*	0.77	0.27	27.86	2.89	0.007	[0.22, 1.31]
*c'*	1.36	2.14	27.92	0.64	0.530	[−3.03, 5.76]
*IND_*belief*_*	5.19	1.80		2.88	0.004	[1.61, 8.68]
**Schaper et al. (****2019****), Experiment 1, JOL**						
*a*	4.01	0.11	6142	37.67	<0.001	[3.80, 4.21]
*b*	0.07	0.06	47.08	1.03	0.310	[−0.06, 0.19]
*c'*	2.82	0.55	88.09	5.11	<0.001	[1.72, 3.92]
*IND_*belief*_*	0.26	0.25		1.02	0.305	[−0.24, 0.76]
**Schaper et al. (****2019****), Experiment 1, JOS**						
*a*	3.71	0.13	6142	29.18	<0.001	[3.46, 3.96]
*b*	0.23	0.10	43.12	2.44	0.019	[0.04, 0.43]
*c'*	7.62	0.90	88.27	8.43	<0.001	[5.83, 9.42]
*IND_*belief*_*	0.86	0.36		2.43	0.015	[0.17, 1.57]
**Schaper et al. (****2019****), Experiment 2, JOL**						
*a*	4.56	0.11	7678	42.52	<0.001	[4.35, 4.77]
*b*	0.03	0.04	118	0.63	0.528	[−0.05, 0.11]
*c'*	2.74	0.43	118	6.44	<0.001	[1.90, 3.58]
*IND_*belief*_*	0.12	0.19		0.63	0.527	[−0.25, 0.49]
**Schaper et al. (****2019****), Experiment 2, JOS**						
*a*	4.44	0.10	7678	42.84	<0.001	[4.23, 4.64]
*b*	0.17	0.09	75.03	1.85	0.069	[−0.01, 0.35]
*c'*	6.86	0.83	115.10	8.31	<0.001	[5.23, 8.50]
*IND_*belief*_*	0.74	0.40		1.84	0.065	[−0.05, 1.53]

*SE, standard error; df, degree of freedom; CI, confidence interval; IND_belief_, the indirect effect of cue on JOLs through beliefs. For Schaper's et al. (2019) Experiment 2, here we only added beliefs (but not self-paced study time) as the mediator variable*.

Our results suggest that the indirect effect of cue on JOLs through beliefs and the direct effect of cue on JOLs were different across various types of cues. Furthermore, the mediation effect of beliefs on JOLs was even different across experiments within the same study (Frank and Kuhlmann, 2017; Su et al., 2018). Comparing the results in the current article and previous studies, we found that the multilevel mediation model could draw similar conclusions as in previous studies, except that the effect of beliefs on JOLs in the multilevel mediation model here did not reach significance in some experiments, which is different from previous studies using other statistical methods such as multilevel moderation model (as we will discuss below) (Frank and Kuhlmann, 2017; Su et al., 2018; Schaper et al., 2019).

#### Comparing the Mediation Effect of Beliefs and Fluency

**Figure 2:**
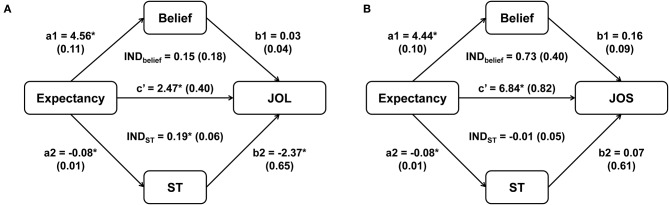
The mediation effect of beliefs and self-paced study time (ST) on the relationship between expectancy and JOLs **(A)** and between expectancy and JOSs **(B)** in Experiment 2 of Schaper et al. (2019). Asterisks indicate significant effects (*p* < 0.05). Standard errors are reported in parentheses.

For Schaper's et al. (2019) Experiment 2, we then added two mediator variables (beliefs and self-paced study time) into one multilevel mediation model, and compared the mediation effect of the two mediators on JOLs/JOSs. The results from the multilevel mediation models for JOLs and JOSs were shown in Figure 2. The indirect effect of expectancy on JOLs through self-paced study time was significant, *IND*_*ST*_ = 0.19, *SE* = 0.06, *z* = 3.23, *p* = 0.001, 95% CI [0.08, 0.31], and study time accounted for 7% of the variance for the expectancy effect on JOLs, suggesting that processing fluency significantly contributed to the expectancy effect on JOLs. However, the mediation effect of beliefs on JOLs was not significant, *IND*_*belief*_ = 0.15, *SE* = 0.18, *z* = 0.82, *p* = 0.410, 95% CI [−0.20, 0.49]. We then compared the mediation effects of study time and beliefs, and found that the two mediation effects did not significantly differ from each other, *Diff*_*IND*_ = 0.04, 95% CI [−0.32, 0.40]. Thus, we did not have enough evidence to support that study time contributed to JOLs more than beliefs. In addition, the direct effect of expectancy on JOLs was significant, *c'* = 2.47, *SE* = 0.40, *t* (113.65) = 6.14, *p* < 0.001, 95% CI [1.68, 3.27], revealing that expectancy could still significantly affect JOLs when the effects of study time and beliefs were controlled (see Figure 2A).

The indirect effect of expectancy on JOSs through beliefs was close to significance, *IND*_*belief*_ = 0.73, *SE* = 0.40, *z* = 1.83, *p* = 0.067, 95% CI [−0.05, 1.51], and beliefs accounted for 10% of the variance for the expectancy effect on JOSs, suggesting that beliefs might play an important role in the formation of JOSs. In contrast, self-paced study time did not significantly mediate the expectancy effect on JOSs, *IND*_*ST*_ = −0.01, *SE* = 0.05, *z* = −0.11, *p* = 0.911, 95% CI [−0.10, 0.09]. The mediation effect of beliefs was higher than that of study time, and this difference was marginally significant, *Diff*_*IND*_ = 0.73, 95% CI [−0.05, 1.53], supporting that beliefs might contribute more to JOSs than study time. The direct effect of expectancy on JOSs was also significant when the effects of beliefs and study time were controlled, *c'* = 6.84, *SE* = 0.82, *t*(120.12) = 8.37, *p* < 0.001, 95% CI [5.22, 8.47], indicating that other factors also significantly contributed to the expectancy effect on JOSs (see Figure 2B).

Our data analysis shows that we can examine the contribution of both beliefs and processing fluency to JOLs in one multilevel mediation model. In addition, we are able to quantitatively compare the effect of beliefs and fluency on JOLs, which has not been analyzed in most of previous studies [for an exception, see Experiment 3 in Yang et al. (2018)].

We should note that the self-paced study time measured in Schaper's et al. (2019) Experiment 2 was not specific to the encoding process for either item or source memory, and might not fully reflect the processing fluency utilized in JOLs or JOSs. In addition, some researchers suggest that self-paced study time may not be an ideal measurement of fluency (Yang et al., 2018). Thus, the non-significant mediation effect of study time on JOSs should not be interpreted as evidence that fluency does not contribute to JOSs because fluency for source memory process might not be precisely measured. We also found that the standard error for the mediation effect of beliefs on JOLs or JOSs was much higher than that for the mediation effect of study time. It is possible that a larger sample size may be needed to more accurately estimate the mediation effect of beliefs and compare the difference between the mediation effects of beliefs and study time. Thus, the non-significant difference between the two mediation effects does not suggest that the contribution of fluency and beliefs to JOLs was exactly the same. The purpose of our data analysis reported here is only to provide an example of how to compare the mediation effect of two mediators in one multilevel mediation model.

## Comparing the Multilevel Mediation and Moderation Model

Some previous studies have built another multilevel linear model, called the multilevel moderation model, to examine the contribution of beliefs (measured with global predictions) to the cue effect on JOLs (Frank and Kuhlmann, 2017; Schaper et al., 2019). For example, Frank and Kuhlmann used multilevel moderation model to examine the role of beliefs in the volume effect on JOLs. Participants were asked to first make global belief-based predictions for words presented in a large and small volume, and then give JOLs to each word during learning. Frank and Kuhlmann calculated the difference in belief-based predictions between large and small volume for each participant, and then performed a multilevel moderation analysis which revealed that this difference in beliefs for each participant significantly moderated the volume effect on JOLs, suggesting an important role of beliefs in the formation of JOLs.

In this section, we first introduce the mathematical equations for the multilevel moderation model, and then examine the similarities and differences between the multilevel mediation and moderation model. Typically, mediation and moderation models address different research questions: while mediation model focuses on whether a mediator variable can account for the relationship between the predictor and outcome variables, moderation model concerns whether a moderator variable can affect the direction or strength of the relationship between another two variables (Baron and Kenny, 1986). However, we will mathematically prove that when we investigate the role of beliefs (measured with global predictions) in JOL process, the two multilevel linear models actually address the same effect of beliefs on JOLs, and multilevel mediation model is more flexible than multilevel moderation model.

### Multilevel Moderation Model

The multilevel moderation model concerns whether participants' beliefs about how much memory performance is affected by different levels of a cue (e.g., large vs. small font size) can moderate the cue effect on JOLs. When we use the multilevel moderation model to measure the contribution of beliefs to JOLs, we first calculate the difference in global belief-based predictions between two levels of the cue for each participant (Frank and Kuhlmann, 2017; Schaper et al., 2019). The equations for the multilevel moderation model are as follows:

(10) $$\begin{array}{l} JOL_{ij}=\beta_{0j}+\beta_{1j}Cue_{ij}+\varepsilon_{ij} \end{array}$$

(11) $$\begin{array}{l} \beta_{0j}=r_{00}+r_{01}DiffBelief_{j}+\mu_{0j} \end{array}$$

(12) $$\begin{array}{l} \beta_{1j}=r_{10}+r_{11}DiffBelief_{j}+\mu_{1j} \end{array}$$

In the equations above, *Cue*_*ij*_ and *JOL*_*ij*_ are the cue level and JOL for each trial, respectively. The *DiffBelief*_*j*_ represents the difference in belief-based predictions between two levels of the cue (e.g., large vs. small font size) for each participant. For example, in the hypothetical dataset from the file *example_data.csv* introduced above, the variable *DiffBelief* should be the difference in global belief-based predictions between Cue Levels 1 and 2, and is equal to 30% for the first participant, 20% for the second participant and 10% for the third participant. We should note that in the multilevel moderation model, we treat *DiffBelief* as a variable at the participant level rather than item level, because there is only one value of *DiffBelief* for each participant, and *DiffBelief* does not vary across trials within a participant. This is different from the variable *Belief* in the multilevel mediation model, which represents the belief-based prediction for each trial and varies across different levels of the cue within a participant. In addition, *r*_00_ and *r*_10_ represent the fixed effects of intercept and the variable *Cue*, respectively. The *r*_01_ and *r*_11_ are the moderation effects of *DiffBelief* on the intercept and the slope of *Cue*. The μ_0*j*_ and μ_1*j*_ represent random effects.

We can substitute Equations (11) and (12) into Equation (10):

(13) $$\begin{array}{l} JOL_{ij}=r_{00}+r_{01}DiffBelief_{j}+r_{10}Cue_{ij}+r_{11}DiffBelief_{j}Cue_{ij}\\ \;+\mu_{0j}+\mu_{1j}Cue_{ij}+\varepsilon_{ij} \end{array}$$

Thus, the multilevel mediation model is actually a multilevel linear regression in which JOLs are regressed on the two main effects (*Cue* and *DiffBelief* ) and their interaction.

In the multilevel moderation model, two effects are of critical interest. One is the interaction between *DiffBelief* and *Cue* (i.e., *r*_11_), which represents whether the difference in belief-based predictions can moderate the effect of cue on JOLs. Another effect of interest is the fixed effect of *Cue, r*_10_, which indicates whether the effect of cue on JOLs is still significant when beliefs are controlled (i.e., whether the cue effect on JOLs could be completely attributed to beliefs).

Similar to the multilevel mediation model, the multilevel moderation effect can be divided into within-participant and between-participant moderation effects. Within-participant moderation effect reflects whether *DiffBelief* can moderate the effect of cue on JOLs for each item within each participant. In contrast, the between-participant moderation effect refers to the moderation effect at the participant level: we can calculate the mean of the variables *Cue* and *JOL* for all trials (including all levels of the cue) separately for each participant, and the between-participant moderation effect represents whether *DiffBelief* moderates the effect of the mean *Cue* on the mean *JOL* across participants (Hofmann and Gavin, 1998). In the studies concerning the contribution of beliefs to JOLs, the within-participant moderation effect is of main interest because we aim to examine whether beliefs contribute to JOLs within each participant. To estimate the within-participant moderation effect, we need to group-mean-center the variable *Cue* to remove the between-participant moderation effect (Hofmann and Gavin, 1998).

### Similarities and Differences Between Two Multilevel Models

According to the mathematical equations, it seems that the multilevel mediation and moderation models concern the effect of beliefs on JOLs in different ways. However, we will mathematically prove that when we investigate the role of beliefs (measured with global predictions) in JOL process, the two models actually address the same effect of beliefs on JOLs, and there are many similarities (but also differences) between the two models.

In the multilevel mediation model [see Equations (1–7)], to identify the within-participant mediation effect of beliefs on the relationship between the cue and JOLs, we should group-mean-center the variables *Cue* and *Belief* for each participant to remove the between-participant mediation effect. For almost (if not all) of the studies on JOLs, the number of to-be-remembered items is the same for different levels of the cue except when there are missing data for some participants (e.g., Hu et al., 2015; Frank and Kuhlmann, 2017; Su et al., 2018; Schaper et al., 2019). When there are only two levels of a cue (Cue Level 1 and Cue Level 2, such as large and small font size) and the number of trials for the two levels is the same, we can simply set the variable *Cue* as 1 for Cue Level 1 and −1 for Cue Level 2, which makes the variable *Cue* group-mean-centered.

To group-mean-center the variable *Belief* , we need to first average the belief-based predictions of all items for each participant and then subtract these means from the belief-based prediction of each item (Vuorre and Bolger, 2018). Suppose that the global belief-based predictions for Cue Levels 1 and 2 are *Belief*_1*j*_ and *Belief*_2*j*_, respectively, for the participant *j*. Because the number of items for Cue Levels 1 and 2 is typically the same, the mean of belief-based prediction for each participant is just equal to (*Belief*_1*j*_ + *Belief*_2*j*_) / 2. To perform the group-mean centering, we should subtract this mean from the variable *Belief* for each trial within the participant *j*. The centered variable *Belief* for trials with Cue Levels 1 and 2 is equal to:

(14) $$\begin{array}{l} Belief_{1j\text{\_}c}=Belief_{1j}-\frac{Belief_{1j}+Belief_{2j}}{2}\\ \;=\frac{Belief_{1j}-Belief_{2j}}{2}=\frac{DiffBelief_{j}}{2} \end{array}$$

(15) $$\begin{array}{l} Belief_{2j\text{\_}c}=Belief_{2j}-\frac{Belief_{1j}+Belief_{2j}}{2}\\ \;=\frac{Belief_{2j}-Belief_{1j}}{2}=-\frac{DiffBelief_{j}}{2} \end{array}$$

in which *Belief*_1*j*_*c*_ and *Belief*_2*j*_*c*_ are the centered belief-based predictions for Cue Levels 1 and 2, and *DiffBelief*_*j*_ is the difference in beliefs between Cue Levels 1 and 2 for participant *j*.

Note that we set the variable *Cue* as 1 for Cue Level 1 and −1 for Cue Level 2. Thus, for the items in the condition of Cue Level 1, the centered belief-based predictions are:

(16) $$\begin{array}{l} Belief_{1j\text{\_}c}=\frac{DiffBelief_{j}}{2}=\frac{DiffBelief_{j}}{2}\cdot Cue_{Level\;1} \end{array}$$

Similarly, for the items in the condition of Cue Level 2:

(17) $$\begin{array}{l} Belief_{2j\text{\_}c}=-\frac{DiffBelief_{j}}{2}=\frac{DiffBelief_{j}}{2}\cdot Cue_{Level\;2} \end{array}$$

Thus, for every trial within participant *j*, the centered belief-based prediction is:

(18) $$\begin{array}{l} Belief_{ij\text{\_}c}=\frac{Diffbelief_{j}}{2}\cdot Cue_{ij} \end{array}$$

In the multilevel mediation model, when JOLs are regressed on the centered variables *Cue* and *Belief* (i.e., path *b* and *c'* in Figure 1), we can substitute Equations (5–7) into Equation (4):

(19) $$\begin{array}{l} JOL_{ij}=r_{00\left(2\right)}+{c^{\prime }Cue}_{ij}+bBelief_{ij\text{\_}c}+\mu_{0j\left(2\right)}+\mu_{c^{\prime }j}Cue_{ij}\\ \;+\mu_{bj}Belief_{ij\text{\_}c}+\varepsilon_{ij\left(2\right)} \end{array}$$

In the equation above, we use the centered variables *Cue* and *Belief* to predict the JOLs at the item level. This equation includes the fixed and random effects for the intercept and the slopes of the variables *Cue* and *Belief* .

Then we can substitute Equation (18) into Equation (19):

(20) $$\begin{array}{l} JOL_{ij}=r_{00(2)}+c^{\prime }Cue_{ij}+\frac{b}{2}DiffBelief_{j}Cue_{ij}+\mu_{0j(2)}+\mu_{c^{\prime }j}Cue_{ij}\\ \;+\frac{\mu_{bj}}{2}DiffBelief_{j}Cue_{ij}+\varepsilon_{ij(2)} \end{array}$$

We can compare the path *b* and *c'* of the multilevel mediation model in Equation (20) with the multilevel moderation model in Equation (13):

(21) $$\begin{array}{l} JOL_{ij}=r_{00}+r_{01}DiffBelief_{j}+r_{10}Cue_{ij}+r_{11}DiffBelief_{j}Cue_{ij}\\ \;+\mu_{0j}+\mu_{1j}Cue_{ij}+\varepsilon_{ij} \end{array}$$

From the equations, we can see that the two models estimate similar effects on JOLs: both models include the fixed effect for the interaction between *DiffBelief* and *Cue*, which reflects the effect of beliefs on the relationship between the cue and JOLs (in the multilevel mediation model, this effect is the same as the effect of the centered variable *Belief* on JOLs, which is path *b*, divided by 2), and the fixed effect for the main effect of variable *Cue* on JOLs when beliefs are controlled. In addition, both models include random intercept and random slope of the variable *Cue*.

Importantly, there are also differences between the two models. Compared with the multilevel mediation model, the multilevel moderation model includes the main effect of *DiffBelief* on JOLs. This main effect reflects the effect of difference in belief-based predictions between Cue Levels 1 and 2 on the mean JOLs for all items (including both Cue Levels 1 and 2) within each participant. In studies on the contribution of beliefs to the cue effect on JOLs, this main effect is typically of no interest. In contrast, compared with the multilevel moderation model, the multilevel mediation model includes the random slope for the interaction between *DiffBelief* and *Cue* (i.e., the random slope for the effect of beliefs on JOLs in the multilevel mediation model). This allows the contribution of beliefs to the cue effect on JOLs to be different across participants.

We should note that although the *DiffBelief* is a variable at the participant level and does not vary across trials within a participant, the variable *Cue* has different values for trials with different cue levels for each participant. In the multilevel mediation model, the variable *DiffBelief* × *Cue* is treated as a variable at the item level [equivalent to the centered variable *Belief* multiplied by 2; see Equation (18)], and has different values in trials with different cue levels for each participant. Thus, the random slope for the effect of the variable *DiffBelief* × *Cue* on JOLs can be added in the multilevel mediation model, which accounts for the variability in the belief effect on JOLs across participants.

It is worth noting that the similarities and differences between the two multilevel models described above are not restricted to the experiments in which the number of items for Cue Levels 1 and 2 is the same for each participant. When there are missing JOL values for one of the cue levels, the number of items for Cue Levels 1 and 2 may be different for some participants. In this case, we can obtain the same mathematical relationship between the multilevel mediation and moderation model (see section “The Relationship Between Multilevel Mediation and Moderation Model When the Trial Numbers for Cue Levels 1 and 2 are Different” in Supplementary Material for the mathematical proof).

According to our discussion above, both the multilevel mediation and moderation models concern the effect of beliefs on JOLs in the same way by examining the interaction between *Cue* and *DiffBelief* . However, the effect of beliefs on JOLs is always fixed across participants in the multilevel moderation model, and we can only estimate the averaged effect of beliefs on JOLs across participants by performing the multilevel moderation analysis. In contrast, in the multilevel mediation model we can add a random slope to account for the variability in belief effect on JOLs across participants. For example, for some participants the difference in belief-based predictions across cue levels may be much smaller than the difference in JOLs, and thus a small change in beliefs can lead to a large amount of change in JOLs. For other participants, the difference in beliefs across cue levels may be higher than the that in JOLs, and a change in beliefs only has a small effect on the JOL value. This variability for belief effect on JOLs across participants may be accounted for by the random slope in the multilevel mediation model.

We should note that the effect of beliefs on JOLs may or may not vary across participants in different empirical datasets, and we may not need to add the random slope for the belief effect on JOLs when performing multilevel mediation analysis for all datasets (such as the hypothetical dataset analyzed in section “Using the Multilevel Mediation Model: A Practical Example”). In addition, it is possible that the variability in the belief effect on JOLs across participants may not be large enough, and thus this random effect cannot be estimated or detected by statistical software such as SPSS. But at the least, multilevel mediation model is more flexible than multilevel moderation model, and the two models should report the same results for the belief effect on JOLs when the random slope for the belief effect on JOLs is not added into the multilevel mediation model.

### Comparing the Two Multilevel Models Fitted to Hypothetical Dataset

In this section, we compare the results from the multilevel mediation and moderation model fitted to the hypothetical dataset in *example_data.csv* introduced in previous sections. When we perform the multilevel mediation analysis for this hypothetical dataset (see section “Using the Multilevel Mediation Model: A Practical Example”), we do not add the random slope for the belief effect on JOLs because this random effect may not exist or cannot be estimated by SPSS. According to our discussion in previous section, we expect that the multilevel moderation and mediation models should provide the same results for the effect of beliefs on JOLs when the random slope for belief effect on JOLs is not added into the multilevel mediation model.

Before performing the multilevel moderation analysis, we need to first group-mean-center the variable *Cue* in order to estimate the within-participant moderation effect, and import the data into SPSS (see section “Using the Multilevel Mediation Model: A Practical Example”). Next, we should create a new variable *DiffBelief* in SPSS, which represents the difference in global belief-based predictions between Cue Levels 1 and 2 for each participant. The value of *DiffBelief* should be equal to 30 for all trials (including both Cue Levels 1 and 2) within the first participant, 20 for the second participant, and 10 for the third participant.

Then we can run the following syntax in SPSS to perform the multilevel moderation analysis:

MIXED JOL WITH Cue_cw DiffBelief 

/FIXED=Cue_cw DiffBelief Cue_cw^*^DiffBelief

/RANDOM=INTERCEPT Cue_cw | SUBJECT(SubID) COVTYPE(DIAG) 

/PRINT=SOLUTION. 

In this syntax, we add the fixed effects for the main effects of *DiffBelief* and the centered cue levels (i.e., *Cue_cw*) on JOLs, and also their interactions. We also add the random intercept for JOLs and random slope for the cue effect on JOLs. In addition, we set the covariance structure as diagonal to remove the correlation between random effects which may make the model fail to converge (as in the multilevel mediation analyses in sections “Using the Multilevel Mediation Model: A Practical Example” and “Fitting Multilevel Mediation Model to Published Data”).

After running this syntax, we can look at the estimates of the fixed effects in the multilevel moderation model, and compare the fixed effects with those from the multilevel mediation model in which beliefs are the only mediator (see section “Using the Multilevel Mediation Model: A Practical Example”). We can see that the regression coefficient for the main effect of cue on JOLs in the multilevel moderation model (β = 10.643) is exactly the same as the cue effect on JOLs (path *c'* in Figure 1) in the multilevel mediation model. In addition, the regression coefficient for the interaction *Cue_cw* × *DiffBelief* in the multilevel moderation model (β = 0.774) is equal to the effect of beliefs on JOLs (path *b* in Figure 1) in the multilevel mediation model divided by 2. These results support our discussion in previous section, suggesting that the multilevel moderation and mediation models provide the same results when the random slope for belief effect on JOLs is not added into the multilevel mediation model.

### Comparing the Two Multilevel Models Fitted to Published Datasets

To further compare the multilevel mediation and moderation model, we fitted both models to published datasets reported in section “Fitting Multilevel Mediation Model to Published Data”. When fitting the multilevel mediation model, we did not add the random slope for the belief effect on JOLs (path *b*), and whether we added other random effects depended on the same criteria reported in previous analyses. For the multilevel moderation model, we added both the random intercept and random slope for the effect of cue on JOLs. Our results are shown in Table 2. We should note that the fixed effect for path *c'* in the multilevel mediation model is exactly the same as the main effect of cue in the multilevel moderation model, and the interaction between *Cue* and *DiffBelief* in the multilevel moderation model is equal to the path *b* in the multilevel mediation model divided by 2 (as in previous section). These results are consistent with our conclusions above, suggesting that the multilevel moderation and mediation models provide the same results for the effect of beliefs on JOLs when the random slope for belief effect on JOLs (path *b*) is not added into the multilevel mediation model.

**Table 2:** Comparison between the multilevel mediation and moderation models fitted to published datasets.

	**Multilevel mediation model**		**Multilevel moderation model**	
	**Path *b***	**Path *c'***	**Main effect of *Cue***	***Cue* × *DiffBelief* interaction**
**Hu et al. (****2015****)**				
Experiment 2	0.33 (0.12)	1.57 (1.12)	1.57 (1.12)	0.16 (0.06)
**Frank and Kuhlmann (****2017****)**				
Experiment 1	0.12 (0.08)	3.60 (1.29)	3.60 (1.29)	0.06 (0.04)
Experiment 2, large-dose group	0.30 (0.07)	3.12 (0.84)	3.12 (0.84)	0.15 (0.03)
Experiment 2, small-dose group	0.18 (0.07)	0.33 (0.78)	0.33 (0.78)	0.09 (0.03)
Experiment 3, large-dose group	−0.03 (0.10)	2.92 (1.04)	2.92 (1.04)	−0.02 (0.05)
Experiment 3, small-dose group	−0.10 (0.06)	2.28 (0.75)	2.28 (0.75)	−0.05 (0.03)
**Su et al. (****2018****)**				
Experiment 2a	0.21 (0.15)	4.26 (1.49)	4.26 (1.49)	0.11 (0.07)
Experiment 2b	0.77 (0.27)	1.36 (2.14)	1.36 (2.14)	0.38 (0.13)
**Schaper et al. (****2019****)**				
Experiment 1, JOL	0.07 (0.06)	2.84 (0.56)	2.84 (0.56)	0.03 (0.03)
Experiment 1, JOS	0.24 (0.09)	7.59 (0.93)	7.59 (0.93)	0.12 (0.04)
Experiment 2, JOL	0.03 (0.04)	2.74 (0.43)	2.74 (0.43)	0.01 (0.02)
Experiment 2, JOS	0.21 (0.08)	6.74 (0.85)	6.74 (0.85)	0.11 (0.04)

*The random slope for the belief effect on JOLs (path b) is not added in the multilevel mediation model. Standard errors are reported in parentheses. For Schaper's et al. (2019) Experiment 2, here we only added beliefs (but not self-paced study time) as the mediator variable*.

We then examined whether adding the random slope for the effect of beliefs on JOLs in the multilevel mediation model could significantly improve the model fit. For each published dataset, we first tried to add the random slope for the belief effect on JOLs into the multilevel mediation model. We found that this random slope could be estimated by SPSS only for five published datasets, including Frank and Kuhlmann‘s (2017) Experiment 1 and small-dose group in Experiment 2, and Schaper's et al. (2019) JOL and JOS conditions in Experiment 1 and JOS condition in Experiment 2. For each of these five datasets, we used Bayesian information criterion (BIC) to compare the fit of the multilevel mediation model with and without the random slope for belief effect on JOLs. The BIC was calculated based on the maximum likelihood (ML) estimation rather than REML estimation because the BIC calculated under REML is generally inappropriate (Gurka, 2006)^3^. We found that adding the random slope for belief effect on JOLs in the multilevel mediation model could significantly improve the model fit for one published dataset, which is the Experiment 1 of Frank and Kuhlmann (2017), ΔBIC = 26.73 (see Table 3 for the BIC from each of the five datasets). This random effect could not be accounted for by the multilevel moderation model.

**Table 3:** Bayesian information criterion (BIC) for the multilevel mediation model with and without the random slope for belief effect on JOLs.

**Experiment**	**Model with random slope for belief effect on JOLs**	**Model without random slope for belief effect on JOLs**
Frank and Kuhlmann (2017), Experiment 1	43551.85	43578.58
Frank and Kuhlmann (2017), Experiment 2, small-dose group	56095.70	56095.47
Schaper et al. (2019), Experiment 1, JOL	97245.94	97236.88
Schaper et al. (2019), Experiment 1, JOS	100038.1	100029.8
Schaper et al. (2019), Experiment 2, JOS	122807.6	122800.8

*For Schaper's et al. (2019) Experiment 2, here we only added beliefs (but not self-paced study time) as the mediator variable*.

Thus, we suggest that future study should decide whether to use multilevel mediation or moderation model to analyze the effect of beliefs on JOLs based on (a) model comparison results about which model can better account for the data (particularly whether adding the random slope for belief effect on JOLs in the multilevel mediation model can significantly improve the model fit), or (b) theoretical hypothesis about whether beliefs may play a mediation or moderation role in JOL process. In addition, it is difficult to directly compare the moderation effect of beliefs and the mediation effect of processing fluency on JOLs. Thus, we should use multilevel mediation model to examine the belief effect on JOLs if we plan to quantitatively compare the contribution of beliefs and fluency on JOLs.

We also note that the results from multilevel moderation model reported here are somewhat different from that reported in previous studies. One important reason for this difference is that when examining the effect of beliefs on JOLs, some previous studies used a multilevel moderation model with only random intercept but not random slope for the cue effect on JOLs (Frank and Kuhlmann, 2017; Schaper et al., 2019), while our model included both the random intercept and random slope. Random slope variation across participants may lead to differences in outcome variable between experimental conditions, creating a spurious effect of experimental conditions in a random-intercept-only model when the true effect might not exist (Barr et al., 2013). Thus, the random-intercept-only model may increase the Type I error rate. Statisticians suggest that we should add all of the random intercepts and slopes into multilevel linear model when the model can converge (Bolker et al., 2009; Barr et al., 2013).

## Inflation of Type I Error in Multilevel Mediation Model

One potential pitfall of using multilevel mediation model to examine the contribution of beliefs to JOLs is the inflation of Type I error when beliefs were measured by global predictions. When we perform multilevel mediation analysis, we need to first regress belief-based predictions on the cue levels, which is similar to examining whether the difference in belief-based predictions between different levels of the cue is significant. In previous studies, this analysis was often performed by a paired *t* test or within-subjects ANOVA at the participant level (Mueller et al., 2014b; Hu et al., 2015; Frank and Kuhlmann, 2017; Su et al., 2018). However, in the multilevel mediation model, we analyze the effect of the cue on beliefs at the item level. In the data at the item level, we treat beliefs as a measurement separately for each trial. When beliefs are measured with global predictions, for each participant the trials within the same level of the cue have the same value for belief-based predictions (as in the hypothetical dataset from *example_data.csv*). Thus, participants' belief-based predictions are replicated many times at the item level, which can inappropriately reduce the standard error and make the cue effect on beliefs easier to be significant, inflating Type I error.

To further illustrate this problem, in the next section, we simulated data for belief-based predictions and compared the Type I and Type II error rates for paired *t* test and multilevel mediation model when investigating the cue effect on beliefs.

### Data Simulation

We simulated data for belief-based predictions from two hypothetical experiments. In each experiment, participants study 40 items with two different levels of a cue (Cue Levels 1 and 2), and each level of the cue contains 20 items. Before the learning phase, they make global belief-based predictions for their memory performance in a later test for Cue Levels 1 and 2 separately (on a percentage scale). In Experiment 1, we assume that all participants' belief-based predictions for both Cue Levels 1 and 2 are randomly generated from the same normal distribution, *N* (50, 10^2^), indicating that there is no difference between the beliefs for Cue Levels 1 and 2 at the group level. In Experiment 2, we assume that participants' belief-based predictions for Cue Level 1 are randomly generated from *N* (60, 10^2^), while their beliefs for Cue Level 2 are generated from *N* (50, 10^2^), indicating that participants believe the items in the condition of Cue Level 1 are easier to remember than those in the condition of Cue Level 2.

We simulated 1,000 datasets for each experiment, and each dataset contained data for belief-based predictions from 30 participants. For each dataset, we first used a paired *t*-test to compare the beliefs for Cue Levels 1 and 2. Then we built a multilevel linear model to use the cue levels to predict the belief-based predictions [see Equations (1–3)]. Similar to the data analyses in previous sections, the variable *Cue* was set as 1 for Cue Level 1 and −1 for Cue Level 2, and the belief-based predictions were group-mean-centered. In addition, we removed the random intercept and random slope for the cue effect on beliefs (as in previous analyses). In Experiment 1, we compared the rate of Type I error (i.e., significant cue effect on beliefs detected by statistical analysis when there is actually no effect) for two methods. In Experiment 2, we compared the rate of Type II error (i.e., failure to detect significant cue effect on beliefs when there is a true effect) for two methods. We provide an example of the simulated datasets in the file *example_sims.xlsx*, which can be downloaded from the folder *simulation* in the OSF repository (https://osf.io/dsnj6/). This file includes a simulated dataset from the hypothetical Experiment 1 and a dataset from Experiment 2, and the results from paired *t* test and multilevel linear model for each dataset.

The simulation results from Experiment 1 revealed that the rate of Type I error for the paired *t*-test was 4.2% (42 out of 1,000 datasets), which is acceptable. However, the Type I error rate for the multilevel linear model was 74.2% (742 out of 1,000 datasets). For all of the datasets in which the *t*-test produced a significant cue effect on beliefs, the multilevel linear model also showed a significant effect. In contrast, for the datasets in which the *t*-test showed a non-significant effect, 73.1% (700 out of 958) of the datasets still revealed a significant cue effect on JOLs from the multilevel linear model. In addition, the results from Experiment 2 indicated that while the rate of Type II error for the paired-sample *t*-test was 3.3% (33 out of 1,000 datasets), the multilevel linear model for all datasets produced a significant cue effect on beliefs. These results indicate that although the multilevel linear model (i.e., path *a* in the multilevel mediation model) showed a lower Type II error rate than the *t* test, the Type I error rate for the multilevel linear model was much higher.

We then look at the example datasets in the file *example_sims.xlsx*, which shows that the global belief-based prediction for each level of the cue is replicated 20 times for each participant in the data at the item level, because there were 20 trials for each cue level within a participant. Comparing the results from the *t*-test and multilevel linear model for each dataset, we can see that the mean difference in belief-based predictions between Cue Levels 1 and 2 across participants (−2.233 and 10.813 for the two simulated datasets) was equal to the fixed effect of cue on beliefs in the multilevel linear model (−1.116 and 5.407 for the two datasets) multiplied by 2, suggesting that the multilevel linear model could accurately detect the overall difference in beliefs between cue levels^4^. However, the standard error in the *t*-test (2.779 and 2.181) was much higher than the standard error for the fixed effect of cue on beliefs in the multilevel linear model (0.216 and 0.170) multiplied by 2, leading to a much higher *t* value in the multilevel linear model. In addition, the degree of freedom for the fixed effect of cue on beliefs in the multilevel linear model (*df* = 1,198) was also much higher than that in the *t*-test (*df* = 29). This is because the belief-based predictions for each participant are replicated many times in the data at the item level, leading to an inappropriate reduction of standard error (and increasing of *df* ) in the multilevel linear model. The higher *t* value in multilevel linear model results in lower *p*-value and an inflation of Type I error.

The inflation of the Type I error should only occur when we regress global belief-based predictions on the cue levels, but not when we regress JOLs on both the cue levels and beliefs. However, the Type I error inflation for the cue effect on beliefs (path *a*) in the multilevel mediation model may lead to an inflation of the Type I error for the indirect effect of cue on JOLs through beliefs [i.e., *IND*_*belief*_; see Equation (8)], which is based on the multiplication of the cue effect on beliefs (path *a*) and the belief effect on JOLs (path *b*) (Bauer et al., 2006). To control the Type I error rate in the multilevel mediation model, we suggest to conduct multilevel mediation analysis only after the paired *t*-test shows a significant effect of the cue on belief-based predictions, which should significantly reduce the Type I error rate when we regress beliefs on the cue at the item level.

## General Discussion

In the current article, we have demonstrated a multilevel mediation model to quantify the contribution of beliefs to a given cue's effect on JOLs. Our examples of fitting the multilevel mediation model to hypothetical and published datasets indicate that it is feasible to use the multilevel mediation model to evaluate the mediation effect of beliefs on the relationship between a cue and JOLs, and quantitatively compare the effect of beliefs and processing fluency on JOLs in one model. We also mathematically prove that while multilevel mediation and moderation models produce similar results about the contribution of beliefs to JOLs, the multilevel mediation model is more flexible because it allows the effect of beliefs on JOLs to vary across participants by adding a random slope.

Our data simulation, however, indicates that compared with the paired *t*-test, multilevel mediation model shows a much higher Type I error rate when we regress the global belief-based predictions on the cue, suggesting that it is necessary to first conduct a *t*-test to determine whether the difference in belief-based predictions between different levels of the cue is significant before implementing the multilevel mediation analysis.

In a nutshell, the following steps are recommended for conducting multilevel mediation analysis to examine the role of beliefs in JOL process: (a) using a paired *t*-test to check if a cue significantly affects belief-based predictions; (b) if yes, group-mean-centering the value for the cue and belief-based predictions to partial out the between-participant variances; (c) performing a multilevel mediation analysis, in which we should remove the random intercept and random slope when the centered variable *Belief* is regressed on the centered variable *Cue* (i.e., path *a*), and add the random intercept for JOLs and random slopes for the effect of the variables *Cue* and *Belief* on JOLs (i.e., path *b* and *c'*) when these random effects can be estimated. In addition, when the processing fluency is also measured for each trial, we can add the group-mean-centered fluency as another mediator variable, and add the random slopes for the cue effect on fluency and the fluency effect on JOLs.

In all of the data analyses reported in this article, we manually group-mean-centered the predictor and mediator variables to emphasize the importance of group-mean centering in the estimation of within-participant mediation effect. But in fact, we do not have to manually group-mean-center the cue levels, belief-based predictions and processing fluency to separate the within-participant and between-participant mediation effect. Instead, we can also build a multilevel mediation model to simultaneously estimate the within-participant and between-participant effect, which can be performed by Mplus or the MLmed macro in SPSS (Preacher et al., 2010; Rockwood and Hayes, 2017). We do not need to center the variables before conducting multilevel mediation analysis with these statistical packages, and can simply use the estimated within-participant mediation effect to represent the contribution of beliefs or fluency to the cue effect on JOLs. We should note that when uncentered mediator variables (beliefs or fluency) are regressed on the cue levels (i.e., path *a* in Figure 1), we should add the random intercept for the mediator variables because the mean of the uncentered mediator variables should vary across participants. In section “Estimating the Within-Participant Mediation Effect With Uncentered Variables: A Practical Example” of the Supplementary Material, we provide a practical example of how to use the MLmed macro to estimate the within-participant mediation effect with uncentered variables.

In our knowledge, there is no golden rule for the requirement of sample size when we use multilevel mediation model to examine the effect of beliefs on JOLs. Generally, statisticians suggest that when we perform multilevel analysis, we should collect data from at least 30 participants with more than 30 trials for each participant, and it may be better to collect data from 50 participants (Hox et al., 2018). A larger sample size may be needed when we aim to detect the difference between the mediation effects of fluency and beliefs on JOLs. In addition, we need to be careful to explain the non-significant difference between the two mediation effects when the standard error for the mediation effects is large.

In the current article, we have mainly discussed the belief judgments measured with global predictions. However, global belief-based predictions measured prior to the learning phase may not fully reflect the beliefs applied to the JOL process because during learning participants may gradually update (or even reverse) their beliefs about how cues affect their memory performance according to their learning experience (Undorf and Erdfelder, 2011; Mueller et al., 2014a). Thus, it may be better to measure item-by-item belief-based predictions during learning, such as pre-study JOLs or JOLs collected in the observer condition (Mueller et al., 2014b; Hu et al., 2015; Price and Harrison, 2017; Yang et al., 2018).

When JOLs and item-by-item belief judgments are collected from the same participants in one experiment (Price and Harrison, 2017; Yang et al., 2018), the multilevel mediation model can be employed to investigate the contribution of beliefs to the cue effect on JOLs with the item-by-item beliefs as the mediator variable (e.g., see Yang et al., 2018). It is worth noting that in this case, the random slope should be included when we regress the belief-based predictions on the cue levels. This is because when the belief judgment for each trial is different, multilevel regression of beliefs on the cue levels with the random slope can converge, which is different from the experiments in which only global belief-based predictions are measured. In addition, when both item-by-item belief judgments and processing fluency are measured for each trial from the same participants, we can also examine whether the effect of processing fluency on JOLs is mediated by beliefs (Yang et al., 2018).

## Data Availability Statement

Publicly available datasets were analyzed in this study. This data can be found here: https://osf.io/dsnj6/.

## Author Contributions

XH, CY, and LL developed the mathematical models. XH and LL analyzed the data from the hypothetical and published datasets, and performed the data simulation. XH, JZ, TF, NS, CY, and LL wrote the manuscript.

### Conflict of Interest

The authors declare that the research was conducted in the absence of any commercial or financial relationships that could be construed as a potential conflict of interest.

## Footnotes

^1^By default, MLmed assumes that the between-participant effect should be estimated for all of the effects in the regression, and all of the random intercepts should be added in the model. We must set */xB* = 0 and */mB* = 0 when there is no between-participant variance for these variables, and */randMint* = 0 when there is no variability across participants in the mean of mediator. Otherwise, MLmed will report an error and the multilevel mediation model cannot be estimated.

^2^One participant in Experiment 2 and one in Experiment 3 of Frank and Kuhlmann (2017) were excluded from data analyses because they offered constant JOLs (i.e., 100) to all items. Because of no variations in JOLs, we removed their data from further analyses, following Frank and Kuhlmann (2017).

^3^We can add a line “/est = ML” in the middle of the syntax for the multilevel mediation model to compute BIC using ML estimation in MLmed.

^4^In the multilevel linear model, we coded Cue Level 1 as 1 and Cue Level 2 as−1, and thus the mean difference in the belief-based predications between Cue Levels 1 and 2 was equal to the fixed effect of cue on beliefs in the multilevel linear model multiplied by 2. If we code Cue Level 1 as 0.5 and Cue Level 2 as −0.5, then the mean difference in beliefs between Cue Levels 1 and 2 is exactly the same as the fixed effect of cue on beliefs in the multilevel linear model.
