# Supplementary material for: Using Multilevel Mediation Model to Measure the Contribution of Beliefs to Judgments of Learning
Source: Front Psychol. 2020 Apr 15;11:637. doi: 10.3389/fpsyg.2020.00637 (PMC7174663; doi:10.3389/fpsyg.2020.00637)
Supplement: Supplementary file 1 [file Data_Sheet_1.docx]

Supplementary Material

# Further explanation about the random slope of path *a* when we regress beliefs on the cue in multilevel mediation model

In order to build a multilevel mediation model to examine the contribution of beliefs to the cue effect on JOLs, we need to first regress the belief predictions for each trial on the cue levels (see Equations (1) – (3) in the main text). In the main text, we also highlight that the random slope of path *a* must be removed to make the model converge when beliefs are measured with global predictions and the predictor variable *Cue* only has two levels (e.g., large and small font size). Here we provide mathematical explanations for the removal of this random slope.

If the random slope of path *a* is added when we regress beliefs on the cue, the equations are as follows:

| ${Belief}_{ij}=\beta_{0j\left( 1 \right)}+a_{j}{Cue}_{ij}+\varepsilon_{ij\left( 1 \right)}$ |  | (1) |
| --- | --- | --- |
| $\beta_{0j\left( 1 \right)}=r_{00\left( 1 \right)}+\mu_{0j\left( 1 \right)}$ |  | (2) |
| $a_{j}=a+\mu_{aj}$ |  | (3) |

When beliefs are measured with global predictions, *Belief_ij_* has the same value for all of the trials within the same level of the cue (e.g., the same font size) for each participant. The intercept *β_0j(1)_* is different across participants due to the random intercept *μ_0j(1)_*. The slope for the variable *Cue* (*a_j_*) is also different across participants when the random slope of *a* (*μ_aj_*) is added into the model.

If the predictor variable *Cue* only has two levels, then for each participant there are only two values for the variable *Cue,* and the variable *Belief* is the same for all trials within the same level of the cue. In addition, the intercept (*β_0j(1)_*) and slope (*a_j_*) are different across participants due to the random effects. In this case, the variable *Belief* for each participant can always be fully accounted for by the linear combination of the intercept, slope and the variable *Cue* for each participant (i.e., *β_0j(1)_* + *a_j_Cue_ij_*), and the residual error *ε_ij_* cannot be estimated because the change in *ε_ij_* can be perfectly mimicked by the change in intercept *β_0j(1)_* and slope *a_j_*. Thus, the model cannot converge when it cannot estimate the residual error.

Let us use the hypothetical dataset in the file *example_data.csv* as an example. The data file *example_data.csv* can be downloaded from the OSF repository (https://osf.io/dsnj6/). In this dataset, the variable *Cue* only has two levels, and we code Cue Level 1 as 1 and Cue Level 2 as -1. When the intercept and slope are different across participants, we can fully account for the variance in the variable *Belief* using the linear combination of the intercept, slope and the variable *Cue* (*Belief* is equal to 15×*Cue*+45 for the first participant, 10×*Cue*+40 for the second participant, and 5×*Cue*+65 for the third participant). Thus, the residual error cannot be estimated for each participant in the regression analysis, which can make the model fail to converge.

When the random slope of *a* (*μ_aj_*) is not added, the slope *a_j_* is constrained to be the same across all participants, and the linear combination (*β_0j(1)_* + *a_j_Cue_ij_*) cannot fully account for the variances of the variable *Belief* for all of the participants even when the cue has only two levels. In the example dataset, when the random slope for path *a* is not added, the slope *a_j_* is equal to 10.38 and should be equal across all participants. In this case, the linear combination (*β_0j(1)_* + *a_j_Cue_ij_*) cannot fully account for the variance in belief-based predictions even when the intercept *β_0j(1)_* is different across participants. Thus, the residual error *ε_ij_* can be estimated and the model can converge.

When beliefs are measured at the item level for each trial (e.g., using pre-study JOLs), the value of the variable *Belief* is different across trials within the same level of the cue for each participant. Thus, the variable *Cue* cannot fully account for the variances in beliefs for each trial, and the residual error *ε_ij_* can be estimated when the random slope of *a* (*μ_aj_*) is added.

# The relationship between multilevel mediation and moderation model when the trial numbers for Cue Levels 1 and 2 are different

In this section, we will mathematically prove that the relationship between multilevel mediation and moderation model described in the main text can be extended to the experiments in which the number of trials for Cue Levels 1 and 2 is different for each participant.

Suppose that for participant *j*, the global belief-based predictions for Cue Levels 1 and 2 are *Belief_1j_* and *Belief_2j_*, and the trial numbers for Cue Levels 1 and 2 are *n_1j_* and *n_2j_*, respectively. We first set the variable *Cue* as 1 for Cue Level 1 and -1 for Cue Level 2 and then group-mean-center the variables *Cue* and *Belief*. The mean of the variable *Cue* for participant *j* is (*n_1j_* - *n_2j_*) / (*n_1j_* + *n_2j_*). Thus, the value of the centered variable *Cue* for Cue Levels 1 and 2 are:

| ${Cue}_{1j\_c}=1-\frac{n_{1j}-n_{2j}}{n_{1j}+n_{2j}}=\frac{2n_{2j}}{n_{1j}+n_{2j}}$ |  | (4) |
| --- | --- | --- |
| ${Cue}_{2j\_c}=-1-\frac{n_{1j}-n_{2j}}{n_{1j}+n_{2j}}=-\frac{2n_{1j}}{n_{1j}+n_{2j}}$ |  | (5) |

The mean of the variable *Belief* for participant *j* is (*n_1j_Belief_1j_* + *n_2j_Belief_2j_*) / (*n_1j_* + *n_2j_*). Thus, the value of the centered variable *Belief* for Cue Levels 1 and 2 are:

| ${Belief}_{1j\_c}={Belief}_{1j}-\frac{n_{1j}{Belief}_{1j}+n_{2j}{Belief}_{2j}}{n_{1j}+n_{2j}}$ $=\frac{n_{2j}\left( {Belief}_{1j}-{Belief}_{2j} \right)}{n_{1j}+n_{2j}}=\frac{n_{2j}}{n_{1j}+n_{2j}}{DiffBelief}_{j}$ |  | (6) |
| --- | --- | --- |
|  |  |  |
| ${Belief}_{2j\_c}={Belief}_{2j}-\frac{n_{1j}{Belief}_{1j}+n_{2j}{Belief}_{2j}}{n_{1j}+n_{2j}}$ $=\frac{n_{1j}\left( {Belief}_{2j}-{Belief}_{1j} \right)}{n_{1j}+n_{2j}}=-\frac{n_{1j}}{n_{1j}+n_{2j}}{DiffBelief}_{j}$ |  | (7) |

in which *DiffBelief_j_* is the difference in beliefs between Cue Levels 1 and 2 for participant *j*.

Then, we can substitute Equations (4)-(5) into Equations (6)-(7). For the items in the condition of Cue Level 1, the centered belief-based predictions are:

| ${Belief}_{1j\_c}=\frac{n_{2j}}{n_{1j}+n_{2j}}{DiffBelief}_{j}=\frac{1}{2}{Cue}_{1j\_c}{Diffbelief}_{j}$ |  | (8) |
| --- | --- | --- |

For the items in the condition of Cue Level 2, the centered belief-based predictions are:

| ${Belief}_{2j\_c}=-\frac{n_{1j}}{n_{1j}+n_{2j}}{DiffBelief}_{j}=\frac{1}{2}{Cue}_{2j\_c}{DiffBelief}_{j}$ |  | (9) |
| --- | --- | --- |

Thus, for every trial within participant *j*, the centered belief-based prediction is:

| ${Belief}_{ij\_c}=\frac{1}{2}{Cue}_{ij\_c}{DiffBelief}_{j}$ |  | (10) |
| --- | --- | --- |

Now, we can substitute the Equation (10) into the multilevel mediation model (see Equation (19) in the main text):

| ${JOL}_{ij}=r_{00\left( 2 \right)}+{c^{'}Cue}_{ij\_c}+\frac{b}{2}{DiffBelief}_{j}{Cue}_{ij\_c}+\mu_{0j\left( 2 \right)}$ $+\mu_{c^{'}j}{Cue}_{ij\_c}+\frac{\mu_{bj}}{2}{DiffBelief}_{j}{Cue}_{ij\_c}+\varepsilon_{ij\left( 2 \right)}$ |  | (11) |
| --- | --- | --- |

When the number of trials for Cue Levels 1 and 2 is different for each participant, we should also group-mean-center the variable *Cue* in the multilevel moderation model to remove the between-participant moderation effect. Thus, the equation for the multilevel moderation model is:

| ${JOL}_{ij}=r_{00}+r_{01}{DiffBelief}_{j}+r_{10}{Cue}_{ij\_c}$ $+r_{11}{Diffbelief}_{j}{Cue}_{ij\_c}+\mu_{0j}+\mu_{1j}{Cue}_{ij\_c}+\varepsilon_{ij}$ |  | (12) |
| --- | --- | --- |

From the equations (11)-(12), we can see that both models include the fixed effects for the interaction between *DiffBelief* and *Cue* (in the multilevel mediation model, this effect is the same as the main effect of the centered variable *Belief* divided by 2) and for the main effect of variable *Cue* on JOLs. In addition, both models include random intercept and random slope of the variable *Cue*.

There are also differences between the two models. Compared with the multilevel mediation model, the multilevel moderation model includes the main effect of *DiffBelief* on JOLs. In contrast, compared with the multilevel moderation model, the multilevel mediation model includes the random slope for the interaction between *DiffBelief* and *Cue*.

# Estimating the within-participant mediation effect with uncentered variables: A practical example

When we perform multilevel mediation analysis with the MLmed macro in SPSS, we can use the original (rather than centered) data for the predictor and mediator variables. The MLmed macro can automatically separate the within- and between-participant variance for each variable, and we can simply use the estimated within-participant effects to represent the mediation effect of beliefs or processing fluency on JOLs. In this section, we provide a practical example of how to estimate the within-participant mediation effect with uncentered variables in the MLmed macro.

Here we perform the analysis on the same hypothetical dataset as in the main text, which is included in the file *example_data.csv* that can be downloaded from the OSF repository (https://osf.io/dsnj6/). We should first import the data into SPSS by selecting the **Import Data – CSV Data** from the **File** menu in SPSS. Then we need to run the syntax *MLMED_Beta_2.sps* in the MLmed package to load the MLmed macro into SPSS.

We first perform a multilevel mediation analysis to examine the mediation effect of beliefs on the relationship between the cue and JOLs. We can open a new syntax window in SPSS and write the following syntax:

MLmed data = DataSet1

/x = Cue

/m1 = Belief

/y = JOL

/cluster = SubID

/randx = 10

/randm = 1

/folder = D:\mlmed_temp\.

The syntax above is very similar to that in the main text except for two differences. First, here we use the original (rather than centered) data for the variables *Cue* and *Belief*. Second, we remove the following lines from the syntax in the main text: */xB* = 0, */mB* = 0 and */randMint* = 0. This is because there should be between-participant variance for the uncentered variables *Cue* and *Belief*, and we need to add the between-participant effect of cue on beliefs and that of beliefs on JOLs. The random intercept for the mediator variable (*Belief*) should also be added into the model due to the variability in the mean of belief-based predictions across participants. In addition, we set */randx* = 10 and */randm* = 1 in the syntax above to remove the random slope for the cue effect on beliefs but add the random slopes for the effect of cue and beliefs on JOLs.

After running this syntax, we receive the following error message:

***Warning: One or more random effect parameters could not be estimated.

Number of random effect parameters that could not be estimated:

2

***********************************************************************

************************** RANDOM EFFECTS ***************************

Level-1 Residual Estimates

Error

Belief .0000

JOL .0000

Random Effect Estimates

Estimate Error

1 9.0659 .0000

2 7.1E+008 1.0000

3 .1111 .0000

4 .0000 1.0000

Random Effect Key

1 Int Belief

2 Int JOL

3 Slope Cue -> JOL

4 Slope Belief -> JOL

***********************************************************************

Note: Indirect Effects Not Calculated Due to Error(s) in Estimated Fixed or Random Effects.

This error message shows that the second and fourth random effect cannot be estimated by SPSS, suggesting these random effects may not exist. Thus, we have to remove these random effects before estimating the parameters in the model. The second random effect is *Int JOL*, which refers to the random intercept for the variable *JOL*. The fourth random effect represents the random slope for the belief effect on JOLs. We should note that the random effects needed to be removed may vary across different empirical datasets. We should start with adding all of the random intercept and random slopes when JOLs are regressed on the cue levels and beliefs, and only remove the random effects reported in the error message.

We then revise the syntax as follows:

MLmed data = DataSet1

/x = Cue

/m1 = Belief

/y = JOL

/cluster = SubID

/randx = 10

/randm = 0

/randYint = 0

/folder = D:\mlmed_temp\.

In the new syntax, we set */randm* = 0 to remove the random slope for the belief effect on JOLs, and */randYint* = 0 to remove the random intercept for the outcome variable (*JOL*). The results from the MLmed are shown in the output window of SPSS after we run this syntax. We first look at the fixed effects:

*************************** FIXED EFFECTS ***************************

***********************************************************************

Outcome: Belief

Within- Effects

Estimate S.E. df t p LL UL

constant 42.5000 2.5000 1.0000 17.0000 .0374 10.7345 74.2655

Cue 10.3571 1.1075 13.0000 9.3522 .0000 7.9646 12.7496

Between- Effects

Estimate S.E. df t p LL UL

Cue 117.5000 22.0436 1.0745 5.3303 .1057 -120.625 355.6253

***********************************************************************

Outcome: JOL

Within- Effects

Estimate S.E. df t p LL UL

constant -1.5000 4.0139 11.0000 -.3737 .7157 -10.3345 7.3345

Cue 10.6429 .7529 1.1422 14.1354 .0319 3.4780 17.8077

Belief 1.5476 .0687 1.0779 22.5347 .0225 .8107 2.2845

Between- Effects

Estimate S.E. df t p LL UL

Cue -213.500 11.2891 11.0000 -18.9120 .0000 -238.347 -188.653

Belief 1.1667 .0943 11.0000 12.3744 .0000 .9592 1.3742

We can see that the within-participant fixed effects reported here are very similar to those in the main text. We then look at the direct and indirect effect of cue on JOLs:

************************* DIRECT EFFECT(S) **************************

Within- Direct Effect

Estimate S.E. df t p LL UL

Cue 10.6429 .7529 1.1422 14.1354 .0319 3.4780 17.8077

Between- Direct Effect

Estimate S.E. df t p LL UL

Cue -213.500 11.2891 11.0000 -18.9120 .0000 -238.347 -188.653

***********************************************************************

************************ INDIRECT EFFECT(S) *************************

Within- Indirect Effect(s)

E(ab) Var(ab) SD(ab)

Belief 16.0289 .0000 .0000

Within- Indirect Effect(s)

Effect SE Z p MCLL MCUL

Belief 16.0289 1.8572 8.6306 .0000 12.4794 19.7800

Between- Indirect Effect(s)

Effect SE Z p MCLL MCUL

Belief 137.0833 28.0791 4.8821 .0000 84.0440 194.5542

Test of Indirect Contextual Effect(s): Between - Within

Dif MCLL MCUL

Belief 121.0544 67.5993 178.9327

The within-participant direct and indirect effects in the current results are consistent with those in the main text.

Next, we add the study time (*ST*) into the model as another mediator, and compare the mediation effect of beliefs and study time on JOLs. The syntax is written as follows:

MLmed data = DataSet1

/x = Cue

/m1 = Belief

/m2 = ST

/y = JOL

/cluster = SubID

/randx = 101

/randm = 01

/randYint = 0

/folder = D:\mlmed_temp\.

This syntax is similar to the syntax in the main text except that we use the original (rather than centered) data for the variables *Cue*, *Belief* and *ST*, and remove the following lines: */xB* = 0, */mB* = 00 and */randMint* = 00. In addition, here we set */randm* = 01 to add the random slope for the effect of study time on JOLs, but remove the random slope for the belief effect on JOLs which cannot be estimated by SPSS (as shown in previous analysis). We also set */randYint* = 0 to remove the random intercept for the variable *JOL* that cannot be estimated in previous analysis.

After running this syntax, we receive the following error message:

***Warning: One or more fixed effect parameters could not be estimated.

Number of fixed effect parameters that could not be estimated:

1

***********************************************************************

*************************** FIXED EFFECTS ***************************

***********************************************************************

Outcome: Belief

Within- Effects

Error

constant 0

Cue 0

Between- Effects

Error

Cue 0

***********************************************************************

Outcome: ST

Within- Effects

Error

constant 0

Cue 0

Between- Effects

Error

Cue 0

***********************************************************************

Outcome: JOL

Within- Effects

Error

constant 0

Cue 0

Belief 0

ST 0

Between- Effects

Error

Cue 0

Belief 0

ST 1

***********************************************************************

Random Effect Key

1 Int Belief

2 Int ST

3 Slope Cue -> JOL

4 Slope Cue -> ST

5 Slope ST -> JOL

***********************************************************************

Note: Indirect Effects Not Calculated Due to Error(s) in Estimated Fixed or Random Effects.

This error message shows that the between-participant effect of the study time (*ST*) on JOLs cannot be estimated by SPSS, suggesting that this between-participant effect may not exist. Thus, we need to remove this effect in the syntax. We should note that whether the between-participant effect exists may vary across different empirical datasets, and we only need to remove the effects reported in the error message.

The syntax should be revised as follows:

MLmed data = DataSet1

/x = Cue

/m1 = Belief

/m2 = ST

/y = JOL

/cluster = SubID

/randx = 101

/randm = 01

/randYint = 0

/mB = 10

/folder = D:\mlmed_temp\.

In this revised syntax, we add */mB* = 10 to keep the between-participant effect of the first mediator (*Belief*) but remove that of the second mediator (*ST*). Then we can run the syntax and examine the results from the MLmed. We first look at the fixed effects:

*************************** FIXED EFFECTS ***************************

***********************************************************************

Outcome: Belief

Within- Effects

Estimate S.E. df t p LL UL

constant 42.5000 2.5000 1.0000 17.0000 .0374 10.7345 74.2655

Cue 10.3571 1.1075 13.0000 9.3522 .0000 7.9646 12.7496

Between- Effects

Estimate S.E. df t p LL UL

Cue 117.5000 22.0436 1.0745 5.3303 .1057 -120.625 355.6253

***********************************************************************

Outcome: ST

Within- Effects

Estimate S.E. df t p LL UL

constant 2.3750 .4917 1.0000 4.8305 .1300 -3.8722 8.6222

Cue -.3388 .0611 1.8863 -5.5439 .0353 -.6175 -.0601

Between- Effects

Estimate S.E. df t p LL UL

Cue 2.4250 4.2625 1.0042 .5689 .6704 -51.1972 56.0472

***********************************************************************

Outcome: JOL

Within- Effects

Estimate S.E. df t p LL UL

constant -1.5000 1.5518 8.5619 -.9666 .3602 -5.0380 2.0380

Cue 7.9273 .7615 1.5422 10.4098 .0206 3.5172 12.3374

Belief 1.6751 .0619 .9006 27.0464 .0321 .6501 2.7002

ST -4.0213 .7964 2.1155 -5.0496 .0330 -7.2745 -.7680

Between- Effects

Estimate S.E. df t p LL UL

Cue -213.500 4.3645 8.5619 -48.9178 .0000 -223.451 -203.549

Belief 1.1667 .0364 8.5619 32.0075 .0000 1.0836 1.2498

The within-participant fixed effects are consistent with those reported in the main text. We then look at the direct and indirect effect of cue on JOLs:

************************* DIRECT EFFECT(S) **************************

Within- Direct Effect

Estimate S.E. df t p LL UL

Cue 7.9273 .7615 1.5422 10.4098 .0206 3.5172 12.3374

Between- Direct Effect

Estimate S.E. df t p LL UL

Cue -213.500 4.3645 8.5619 -48.9178 .0000 -223.451 -203.549

***********************************************************************

************************ INDIRECT EFFECT(S) *************************

Within- Indirect Effect(s)

E(ab) Var(ab) SD(ab)

Belief 17.3497 .0000 .0000

ST 1.3623 .1706 .4131

Within- Indirect Effect(s)

Effect SE Z p MCLL MCUL

Belief 17.3497 1.9641 8.8333 .0000 13.4809 21.2171

ST 1.3623 .3681 3.7004 .0002 .7118 2.1516

Between- Indirect Effect(s)

Effect SE Z p MCLL MCUL

Belief 137.0833 26.0841 5.2554 .0000 85.7017 188.7801

***********************************************************************

Within- Indirect Effect Contrasts

Dif MCLL MCUL

ab2-ab1 -15.9874 -19.8757 -12.1701

Test of Indirect Contextual Effect(s): Between - Within

Dif MCLL MCUL

Belief 119.7336 68.4989 171.2830

We can see that the within-participant direct and indirect effects also replicate those in the main text.
